# Machine learning-based prediction of *E. coli* infection in hospitalized patients using a no-code analytical framework

**DOI:** 10.1038/s41598-026-59795-y

**Published:** 2026-07-04

**Authors:** Mona Gharib, Mahmoud E. F. Abdel-Haliem, Nagham M. Nassar

**Affiliations:** 1 Department of Mathematics, Faculty of Science, Zagazig University, Zagazig, 44519 Egypt; 2 Department of Botany and Microbiology, Faculty of Science, Zagazig University, Zagazig, 44519 Egypt

**Keywords:** Machine learning, Risk assessment, Data mining, Artificial intelligence, No-code ML, Clinical prediction, Computational biology and bioinformatics, Diseases, Health care, Medical research, Microbiology

## Abstract

Hospital-acquired infections (HAIs) remain a major global concern, contributing significantly to increased morbidity, mortality, and healthcare costs. Among the causative pathogens, *Escherichia coli* (*E. coli*) is one of the most frequently isolated microorganisms, particularly in urinary tract infections (UTIs), bloodstream infections, and surgical site infections. Early and accurate prediction of *E. coli* infection in hospitalized patients remains a significant clinical challenge, yet it has the potential to substantially improve patient outcomes. In addition, identifying patient-related risk factors can support targeted infection control strategies. This study aims to evaluate a no-code machine learning (ML) approach for early prediction of *E. coli* infection and to identify associated risk factors. ML techniques provide a powerful alternative by enabling the analysis of high-dimensional and heterogeneous datasets, facilitating the discovery of hidden patterns and supporting individualized risk prediction. In this study, a total of 300 clinical samples was collected as a training dataset from hospitalized patients between July 2024 and February 2025 across multiple units of Zagazig University Hospital, Sharkia, Egypt. An independent internal validation dataset of 100 samples was collected during May 2026 from the same hospital, its purpose was to evaluate model generalizability on completely unseen data. Bacterial isolates were identified using standard biochemical methods. Data analysis was performed using the Orange visual programming platform, implementing a modular ML pipeline that integrates data preprocessing, feature handling, model training, and performance evaluation within a no-code environment. The Naive Bayes model, shows potential for predicting *E. coli* infection in hospitalized patients. The model is intended to predict *E. coli* infection at the time of specimen collection, before culture results are finalized, depending on clinical data. However, further validation in larger, multi-center prospective cohorts is needed before clinical implementation.

**Supplementary Information:**

The online version contains supplementary material available at 10.1038/s41598-026-59795-y.

## Introduction

The incidence of *E. coli*-associated infections, including (UTIs), intra-abdominal infections, bacteremia, and sepsis, continues to rise, with antimicrobial resistance playing a critical role in this trend^1,2^. The predominance of *E. coli* in healthcare environments is largely attributed to its natural presence in the human gastrointestinal tract, its ability to acquire and disseminate antibiotic resistance genes, and its persistence on hospital surfaces and medical equipment^1,3^. Furthermore, the emergence of extended-spectrum beta-lactamase (ESBL)-producing strains has significantly limited treatment options and reinforced the need for continuous surveillance and infection control strategies^4^.

Traditional methods for hospital infection surveillance depending on manual chart reviews and culture-based pathogen identification, which are time-consuming, labor-intensive, and prone to underreporting^5^. This often delays targeted antibiotic therapy and contributes to increased morbidity, mortality, and healthcare costs. In recent years, ML has emerged as a powerful tool to support infection prevention and control programs by enabling early prediction of infections using routine clinical data available at the time of specimen collection^6–8^. ML models can identify high-risk patients before culture results are finalized, allowing clinicians to initiate timely empirical therapy, reduce unnecessary broad-spectrum antibiotic use, and support antimicrobial stewardship efforts^6–9^. Various ML algorithms have been successfully applied to infection prediction tasks, each with distinct strengths. Random Forest and Neural Networks have demonstrated strong predictive performance in healthcare-associated infection (HAI) detection, with some models achieving AUC values exceeding 0.90 for surgical site infection prediction and significantly improving detection rates when integrated with electronic health records^7,8^. Naive Bayes classifiers, despite their simplifying assumption of conditional independence, remain widely used for their computational efficiency and robustness, particularly in settings with limited data^10^. Despite these promising applications, the adoption of ML-based surveillance systems in routine clinical practice remains limited, particularly in low-resource settings^5^. Recent studies have successfully applied ML models to predict infections^11^, sepsis^12^, and organ dysfunction in critically ill patients^13^, often outperforming traditional approaches. From a computational perspective, these advancements align with the growing adoption of Automated Machine Learning (AutoML) frameworks, which aim to simplify model development while maintaining high predictive performance^14^. In addition, the emergence of human-centered artificial intelligence emphasizes the importance of usability, interpretability, and accessibility, particularly in clinical environments where domain experts may lack advanced programming expertise^15^.

Orange Data Mining is an open-source, no-code data analytics platform that enables end-to-end ML through a visual workflow interface^16^. Its graphical user interface emphasizes exploratory data analysis (EDA) and rapid model prototyping, allowing users to construct modular analytical pipelines without requiring extensive coding knowledge^17^. This visual approach enhances transparency, reproducibility, and ease of use, making it particularly suitable for interdisciplinary applications that bridge clinical and computational domains. Moreover, such platforms support the development of interpretable models, which is a critical requirement in medical applications where understanding model behavior is essential for trust and adoption^18^. Despite these advancements, challenges remain in ensuring model generalizability and avoiding overfitting, particularly when working with limited or highly structured clinical datasets. High predictive performance may sometimes reflect dataset-specific patterns rather than true generalizable relationships, highlighting the importance of robust validation strategies^19^. This study aims to highlight the importance of ML in early prediction of HAIs, using *E. coli* as a clinical model. Through a hospital-based survey, we first identify the epidemiological prevalence patterns of *E. coli*, then evaluate a no-code ML approach (Orange platform version 3.40) to demonstrate how such tools can support infection control teams. By using accessible platforms, we seek to bridge the gap between advanced analytics and clinical practice, empowering healthcare professionals, regardless of coding expertise, to adopt predictive surveillance for faster, data-driven decisions.

## Results

### Samples collection

**Fig. 1:**
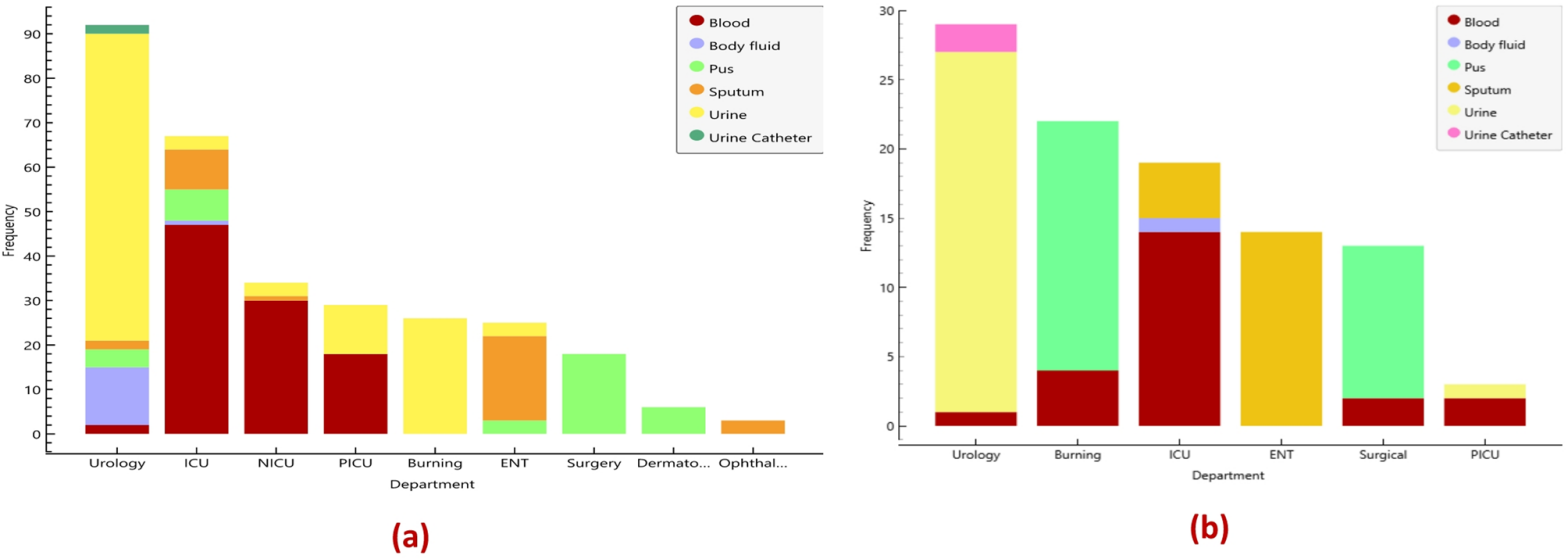
Distribution of specimen types across different hospital departments for (**a**) the training dataset (*n* = 300), and (**b**) the internal validation dataset (*n* = 100).

**Fig. 2:**
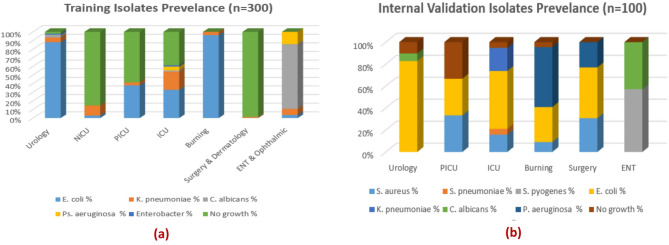
The stacked column shows the prevalence of bacterial isolates in (**a**) the training dataset (*n* = 300), and (**b**) Internal Validation Dataset (*n* = 100) collected from various departments.

The study used two separate datasets. The training dataset consisted of 300 samples collected between July 2024 and February 2025, which were used for model development and 10-fold stratified cross-validation. An independent internal validation dataset consisted of 100 samples collected during May 2026, which were kept completely separate (see Supplementary Table S1-S8). Among the 300 training samples, Urology (*n* = 92, 30.6%), Neonates Intensive Care Unit (NICU) (*n* = 34, 11.3%), Pediatric Intensive Care Unit (PICU) (*n* = 29, 9.7%), Intensive Care Unit (ICU) (*n* = 67, 22.3%), Burning (*n* = 26, 8.7%), surgery and dermatology (*n* = 24, 8%), finally, Ear, Nose and Throat (ENT) and Ophthalmic (*n* = 28, 9.3%). The training data population included 51.67% males and 48.33% females, with ages ranging from 1 day to 87 years. While, the internal validation dataset (*n* = 100) included patients from multiple departments: Urology (*n* = 29, 29%), Burning unit (*n* = 22, 22%), ICU (*n* = 19, 19%), ENT (*n* = 14, 14%), Surgery (*n* = 13, 13%), and PICU (*n* = 3, 3%). The population was 31% male and 69% female, aged 8–72 years. The distribution of specimen types varied across departments as shown in Fig. 1. In the training dataset, blood samples were the most frequently collected specimen type in critical care units, including ICU, PICU and NICU (*n* = 47, 70.15%, *n* = 18, 62.07% and *n* = 30, 88.24%, respectively). In contrast, urine samples predominated in the Urology department (*n* = 69, 75%) and the Burn unit (*n* = 26, 100%), which is consistent with the higher incidence of urinary tract infections in these settings. While, In the internal validation dataset (*n* = 100), Urine samples were the most frequent in Urology (*n* = 26), pus was the predominant specimen in both Burn (*n* = 18) and Surgery (*n* = 11). Blood samples were the most common in ICU (*n* = 14), while sputum was the only specimen in ENT (*n* = 14). The variation in specimen distribution across departments highlights the heterogeneity of clinical conditions and supports the need for department-specific analytical and predictive modeling approaches, Fig. 2.

### Microbial identification

All isolates were then subjected to Gram staining and further characterized by standard biochemical tests as shown in (Fig. 8) with confirmation by the VITEK 2 automated system (bioMérieux, France). the identification scheme included Gram staining, motility testing, and a panel of biochemical reactions: indole production, citrate utilization, urease activity, oxidase test, methyl red (MR), and Voges Proskauer (VP) tests. The characteristic reactions for each isolate are summarized in Table 1.

**Table 1:** Biochemical results of bacterial isolates identification:.

Biochemical test	*S. aureus*	*S. pneumoniae*	*S. pyogenes*	*E. coli*	*K. pneumoniae*	Enterobacter	*P*. aeruginosa
Gram stain	+ve Cocci	+ve Cocci	+ve Cocci	-ve Bacilli	-ve Bacilli	-ve Bacilli	-ve Bacilli
Catalase	+ve	-ve	-ve	+ve	+ve	+ve	+ve
Coagulase	+ve	ND	ND	ND	ND	ND	ND
Hemolysis	Beta	Alfa	Beta	Variable	Gamma	Gamma	Beta
Optochin	ND	+ve	-ve	ND	ND	ND	ND
Bacitracin	ND	ND	+ve	ND	ND	ND	ND
Motility	Non motile	Non motile	Non motile	Motile	Non motile	Motile	Motile
Indole	-ve	ND	ND	+ve	-ve	-ve	-ve
Citrate	+ve	ND	ND	-ve	+ve	+ve	+ve
Urease	+ve	-ve	-ve	-ve	+ve	+ve	-ve
Oxidase	-ve	-ve	-ve	-ve	-ve	-ve	+ve
MR	ND	ND	ND	+ve	-ve	-ve	-ve
VP	+ve	-ve	-ve	-ve	+ve	+ve	-ve

The distribution of bacterial and fungal isolates, along with the prevalence of *E. coli*, is summarized in Tables 2 and 3 for training and internal validation data. For rapid visual comparison of microbial prevalence across departments, a stacked bar chart was performed. The in the training data, overall prevalence of *E. coli* was 47.3% (142/300), making it the most frequently isolated microorganism, followed by *K. pneumoniae* (13%, 39/300) and *C. albicans* (8.7%, 26/300). Notably, no growth was observed in 28% (84/300) of specimens, with the highest rates in NICU (85.3%) and PICU (58.6%). Department-specific distribution patterns revealed substantial heterogeneity in microbial prevalence. *E. coli* was highly dominant in the Urology (88%) and Burn units (96.2%), consistent with the clinical association of these departments with urinary and wound infections. In contrast, *K. pneumoniae* was more prevalent in the Surgery unit (50%), while *C. albicans* was predominantly observed in ENT cases (75%). These findings highlight significant variability in microbial profiles across hospital units, suggesting that infection patterns are context-dependent. Such heterogeneity provides a strong foundation for applying ML models to capture department-specific patterns and improve predictive performance.

**Table 2:** Prevalence of Bacterial Isolation in the Training Dataset from Each Hospital Unit:.

Unit	Total samples	*E. coli*		*K. pneumoniae*		*C. albicans*		*P*. aeruginosa		Enterobacter		No growth	
		No	%	No	%	No	%	No	%	No	%	No	%
Urology	92	81	88	5	5.4	3	3.3	-	0	1	1.1	2	2.2
NICU	34	1	2.9	4	11.8	-	-	-	0	-	0	29	85.3
PICU	29	11	37.9	1	3.5	-	-	-	0	-	0	17	58.6
ICU	67	22	32.8	14	20.9	1	1.5	3	4.5	1	1.5	26	38.8
Burning	26	25	96.2	1	3.8	-	-	-	0	-	0	-	0
Surgery & Dermatology	24	1	4.2	12	50	1	4.2	-	0	-	0	10	41.7
ENT & Ophthalmic	28	1	3.6	2	7.1	21	75	4	14.3	-	0	-	0
Total	300	142	47.3	39	13	26	8.7	7	2.3	2	0.7	84	28

In the internal validation dataset (*n* = 100), a total of 29 samples were collected from the Urology department, of which 24 (82.76%) were positive for *E. coli*, 2 for *C. albicans*, and 3 showed no growth. In the Burn unit (*n* = 22), *P. aeruginosa* was the most frequently isolated pathogen (*n* = 12, 54.55%), followed by *E. coli* (*n* = 7, 31.82%), *S. aureus* (*n* = 2, 9.09%), and one sample with no growth (4.55%). In the ICU (*n* = 19), *E. coli* was the predominant isolate (*n* = 10, 52.63%), followed by *K. pneumoniae* (*n* = 4, 21.05%), *S. aureus* (*n* = 3, 15.79%), *S. pneumoniae* (*n* = 1, 5.26%), and one sample with no growth (5.26%). In the ENT department (*n* = 14), *S. pyogenes* was isolated from 8 samples (57.14%) and *C. albicans* from 6 samples (42.86%). In the Surgical department (*n* = 13), *E. coli* was the most common isolate (*n* = 6, 46.15%), followed by *P. aeruginosa* (*n* = 3, 23.08%), while 4 samples (30.77%) showed no growth. In the PICU (*n* = 3), one sample was positive for *E. coli* (33.33%), one for *S. aureus* (33.33%), and one showed no growth (33.33%). The validation dataset contained 48 *E. coli*-positive (48%) and 52 *E. coli*-negative (52%) samples, providing a balanced evaluation cohort.

### Data analytics and machine learning modeling

Data analysis was conducted using the Orange Data Mining platform (version 3.40) through a structured visual workflow (Fig. 7), where interconnected widgets were used for data preprocessing, modeling, and visualization. Widgets accept data as their input and display or send results as their output.

#### Visualization and graphical analysis

**Fig. 3:**
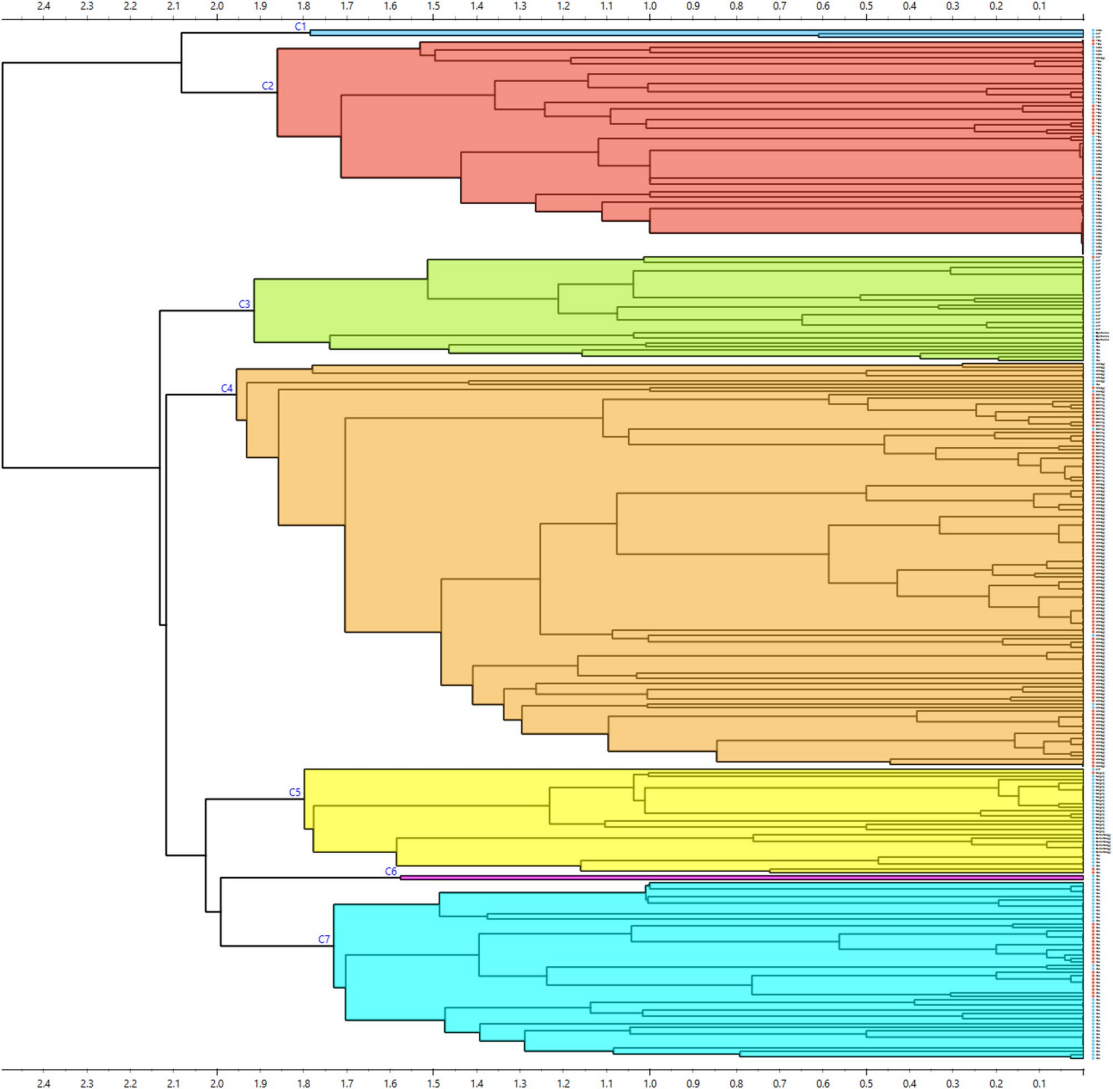
Hierarchical clustering dendrogram of the training dataset (*n* = 300) showing 7 distinct clusters (C1–C7). The analysis was based on patient demographics (age, gender), specimen types, microbial isolates, and hospital departments. PICU and NICU units form a close cluster at the top, burning unit and Urology departments cluster together in the middle, while ICU, Dermatology and Surgery units form a separate cluster at the bottom, suggesting similarities in their clinical and microbiological profiles.

The resulting dendrogram, shown in Fig. 3, illustrates the clustering patterns of departments in the training samples based on *E. coli* prevelance. Departments with similar characteristics such NICU and PICU which characterized by high rates of no-growth specimens (85.3% and 58.6%, respectively), while Urology and Burn Unit cluster together (both showing high *E. coli* prevalence (88% and 96.2%, respectively,). ENT and Ophthalmology share a branch, consistent with their higher rates of *C. albicans*.

**Fig. 4:**
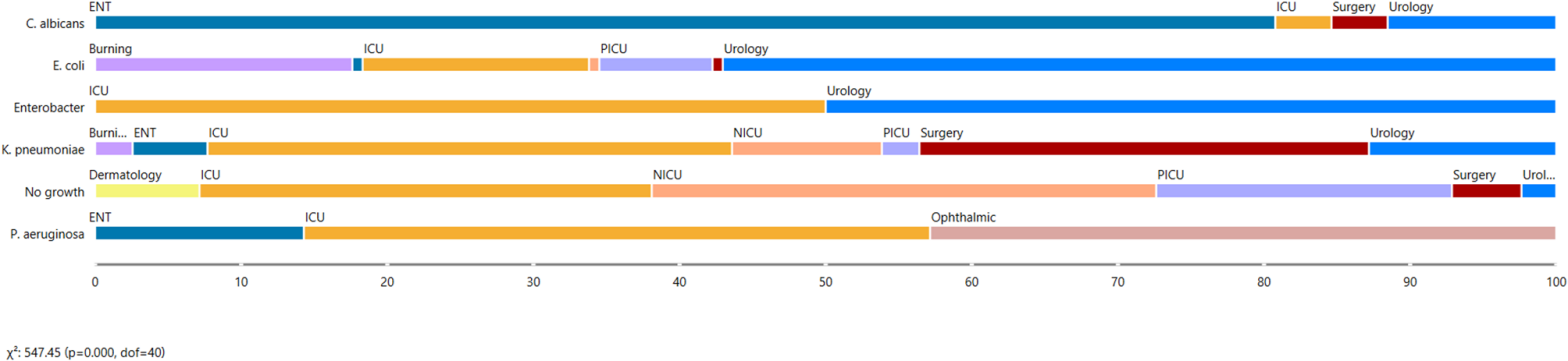
The box plot analysis was generated on training data using Orange, which automatically performed a Chi-square test to evaluate the significance of differences in microbial prevalence across departments. The plot demonstrates the variability in microbial prevalence across departments. The Chi-square test (χ² = 547.45, df = 40, *p* < 0.001) indicates statistically significant differences between departments, aligning with the clustering patterns observed in Fig. 3.

To assess the statistical significance of differences in microbial prevalence in the training data across hospital departments, a Chi-square test was performed. The results showed a highly significant difference (χ² = 547.45, degrees of freedom (df) = 40, *p* < 0.001), indicating that microbial distribution varies substantially between departments. Importantly, this result reflects significant variation rather than independence, suggesting that each department has a unique microbial composition influenced by clinical practices, patient populations, and specimen types. These findings reinforce the need for department-specific infection control and predictive modeling strategies. The variability in microbial distribution is further illustrated in Fig. 4, which visually highlights differences across departments.

#### Feature importance and predictive factors (explainability)

**Fig. 5:**
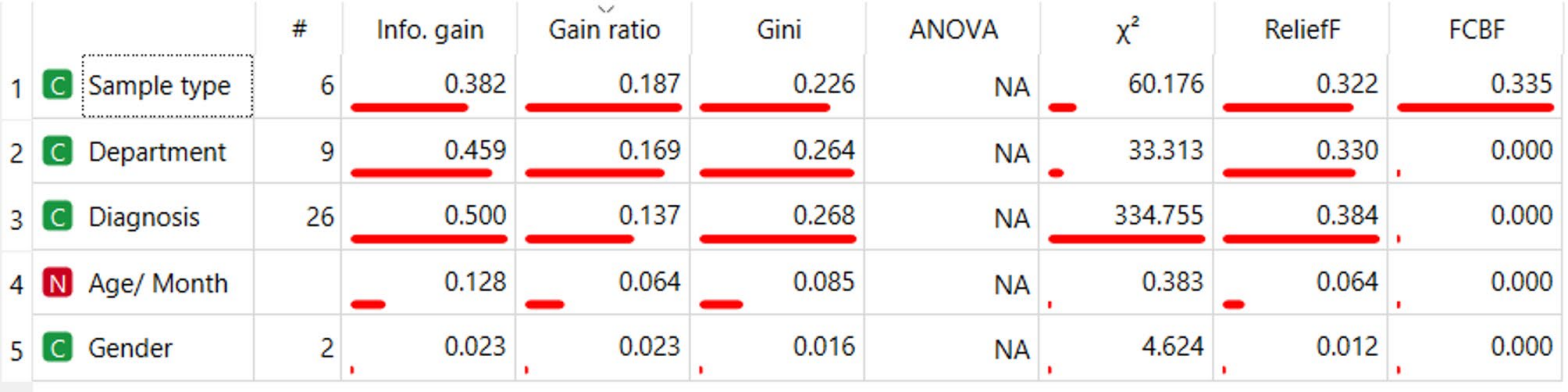
Feature ranking results for *E. coli* prediction in the training samples. Variables are ranked by their importance in predicting *E. coli* infection using multiple metrics: Information Gain: the expected amount of information (reduction of entropy), Gain Ratio: a ratio of the information gain and the attribute’s intrinsic information, which reduces the bias towards multivalued features that occurs in information gain, Gini: the inequality among values of a frequency distribution, ANOVA: the difference between average values of the feature in different classes, Chi2: dependence between the feature and the class as measured by the chi-square statistic, ReliefF: the ability of an attribute to distinguish between classes on similar data instances, and, FCBF (Fast Correlation Based Filter): entropy-based measure, which also identifies redundancy due to pairwise correlations between features.

To identify the most influential predictors of *E. coli* infection in the training data, feature ranking analysis was performed using multiple scoring methods, including Information Gain, Gain Ratio, Gini Index, Chi-square, ReliefF, and Fast Correlation-Based Filter (FCBF). The results, presented in Fig. 5, rank the variables according to their contribution to predicting *E. coli* positivity. The feature ranking analysis confirms that Sample type, Department, and Diagnosis are the strongest predictors of *E. coli* infection. This aligns with the logistic regression model, which identified urine specimens (coefficient = 1.5, *P* < 0.001) and the Urology department (coefficient = 1.2, *P* < 0.01) as significant predictors. This agreement between statistical modeling and feature ranking enhances the interpretability and reliability of the predictive framework. Moreover, these results align with the clustering patterns observed in Fig. 3, where Urology and Burn Unit both with high *E. coli* prevalence formed a distinct cluster.

#### Machine learning model performance

To assess generalizability, we performed internal validation using an independent dataset of 100 samples collected during May 2026 from the same institution. These samples were not used in model training or hyperparameter tuning. All classifiers were applied to this internal dataset without any re-training. Table 3 compares model performance between 10-fold stratified cross-validation using training data (*n* = 300) and internal validation (*n* = 100). For internal validation, 95% confidence intervals for Area Under the Curve (AUC) were calculated using bootstrap resampling with 1,000 iterations. For threshold-dependent metrics (accuracy, F1, precision, recall, and MCC), the default classification threshold of 0.5 was applied^20^.

**Table 3:** Comparison of all classifiers between 10-fold stratified cross-validation on training data (*n* = 300) and internal validation dataset (*n* = 100).

Model	Training Dataset (*n* = 300)						Internal Validation Dataset (*n* = 100)					
	AUC	CA	F1	Prec	Recall	MCC	AUC	CA	F1	Prec	Recall	MCC
kNN	0.804	0.723	0.715	0.698	0.732	0.447	0.556	0.600	0.545	0.600	0.500	0.196
Constant	0.500	0.527	0.000	0.000	0.000	0.000	0.500	0.520	0.000	0.000	0.000	0.000
Logistic regression	0.879	0.823	0.810	0.825	0.796	0.645	0.806	0.730	0.710	0.733	0.688	0.459
Neural Network	0.941	0.903	0.899	0.890	0.908	0.806	0.782	0.760	0.739	0.773	0.708	0.519
Random Forest	0.952	0.893	0.887	0.887	0.887	0.786	0.770	0.710	0.695	0.702	0.688	0.419
Naive Bayes	0.940	0.853	0.838	0.877	0.803	0.707	0.843	0.750	0.737	0.745	0.729	0.499
AdaBoost	0.900	0.873	0.867	0.861	0.873	0.746	0.726	0.730	0.710	0.733	0.688	0.459

AUC, Area Under the Curve (measure of discriminative ability); CA, Classification Accuracy (proportion of correct predictions); F1, F1 score (harmonic mean of precision and recall); Prec, Precision (positive predictive value); Recall, Sensitivity (true positive rate); MCC, Matthews Correlation Coefficient (measure of the quality of binary classifications, ranging from − 1 to + 1).

**Fig. 6:**
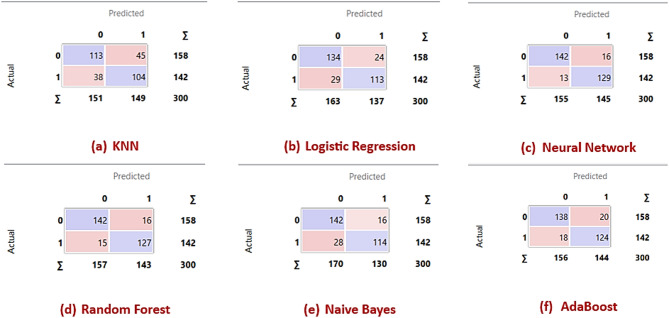
Confusion matrices for all models on the training dataset (*n* = 300). Each matrix shows TP, TN, FP, and FN counts for (**a**) kNN, (**b**) Logistic Regression, (**c**) Neural Network, (**d**) Random Forest, (**e**) Naive Bayes, (**f**) AdaBoost.

As shown in Table 3, Random Forest achieved the highest performance with an AUC of 0.952 on training dataset, followed by Neural Network (AUC = 0.941) and Naive Bayes (AUC = 0.940, while K-Nearest Neighbors (kNN) (AUC = 0.804) showed lower performance. On internal validation using 100 independent samples, we calculated 95% confidence interval (CI). Naive Bayes achieved the highest AUC of 0.843 (95% CI: 0.762–0.924), outperforming all other models. Logistic Regression (AUC = 0.806, 95% CI: 0.718–0.894) and Neural Network (AUC = 0.782, 95% CI: 0.688–0.876) also showed good generalizability. In contrast, Random Forest dropped from an AUC of 0.952 on training dataset to an AUC of 0.770 (95% CI: 0.675–0.865) on internal validation dataset, while AdaBoost dropped from 0.900 to 0.726 (95% CI: 0.625–0.827). These drops suggest that more complex models such as Random Forest and AdaBoost experienced mild overfitting when trained on our limited sample size (*n* = 300). Notably, Naive Bayes showed the smallest performance drop (0.940 to 0.843) and achieved the highest internal validation AUC, indicating better generalizability for this dataset. This finding suggests that for limited sample sizes, simpler models may be more reliable for clinical prediction tasks. The performance of the developed ML models for predicting *E. coli* infection was evaluated using the confusion matrix obtained from the training dataset (*n* = 300). The confusion matrix was used to determine the number of correctly and incorrectly classified samples, including true positive, true negative, false positive, and false negative predictions. Based on these values, different performance indicators such as accuracy, sensitivity, and specificity were calculated to assess the ability of each model to distinguish between infected and non-infected cases. The obtained evaluation results for the different ML algorithms are summarized in Table 4 and Fig. 6.

**Table 4:** Performance Evaluation of ML Models for Prediction of *E. coli* Infection using the Training Dataset (*n* = 300).

Model	TN	FP	FN	TP	Accuracy	Sensitivity	Specificity
kNN	113	45	38	104	72.3%	73.2%	71.5%
Logistic regression	134	24	29	113	82.3%	79.6%	84.8%
Neural Network	142	16	13	129	90.3%	90.8%	89.9%
Random Forest	142	16	15	127	89.7%	89.4%	89.9%
Naive Bayes	142	16	28	114	85.3%	80.2%	89.9%
AdaBoost	138	20	18	124	87.3%	87.3%	87.3%

TN True Negative, FP False Positive, FN False Negative, TP True Positive.

Table 4 summarizes the confusion matrix components and derived performance metrics for all models on the training dataset (*n* = 300). Among all classifiers, Neural Network achieved the highest accuracy (90.3%), sensitivity (90.8%), and specificity (89.9%), followed closely by Random Forest (accuracy: 89.7%, sensitivity: 89.4%, specificity: 89.9%). Naive Bayes demonstrated moderate performance with an accuracy of 85.3%, sensitivity of 80.2%, and specificity of 89.9%. Logistic Regression achieved an accuracy of 82.3%, sensitivity of 79.6%, and specificity of 84.8%. AdaBoost showed balanced performance with accuracy, sensitivity, and specificity all at 87.3%. While, kNN had the lowest performance among the evaluated classifiers, with an accuracy of 72.3%, sensitivity of 73.2%, and specificity of 71.5%. These findings indicate that Neural Network and Random Forest provided the most accurate classification on the training dataset.

**Fig. 7:**
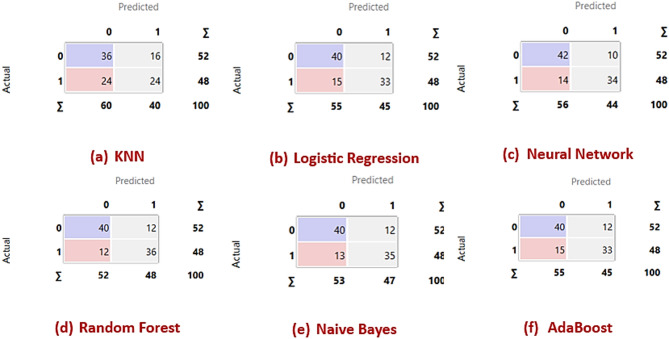
Confusion matrices for all models on the validation dataset (*n* = 100). Each matrix shows TP, TN, FP, and FN counts for (**a**) kNN, (**b**) Logistic Regression, (**c**) Neural Network, (**d**) Random Forest, (**e**) Naive Bayes, (**f**) AdaBoost.

To ensure the reliability of our models and evaluate their predictive ability on new data, we generated confusion matrices for all classifiers on the internal validation dataset (Table 5 and Fig. 7). These matrices provided accuracy, sensitivity, and specificity metrics, which were then compared with the corresponding training data results. This comparison was essential to assess whether the models maintain their performance on unseen data or exhibit overfitting to the training set.

**Table 5:** Performance Evaluation of ML Models for Prediction of *E. coli* Infection using the Internal Validation Dataset (*n* = 100).

Model	TN	FP	FN	TP	Accuracy	Sensitivity	Specificity
kNN	36	16	24	24	60%	50%	69.2%
Logistic regression	40	12	15	33	73%	68.8%	76.9%
Neural Network	42	10	14	34	76%	70.8%	80.8%
Random Forest	40	12	12	36	76%	75%	76.9%
Naive Bayes	40	12	13	35	75%	72.9%	76.9%
AdaBoost	40	12	15	33	73%	68.8%	76.9%

TN True Negative, FP False Positive, FN False Negative, TP True Positive.

Table 5 summarizes the confusion matrix components and derived performance metrics for all classifiers on the internal validation dataset (*n* = 100). Among all models, Neural Network and Random Forest achieved the highest accuracy (76.0% for both). Neural Network also showed the best specificity (80.8%), while Random Forest demonstrated the highest sensitivity (75.0%). Naive Bayes showed the smallest performance drop (from 85.3% to 75.0%), indicating better stability and generalizability. Logistic Regression and AdaBoost showed comparable performance (accuracy: 73.0%, sensitivity: 68.8%, specificity: 76.9%). Finally, kNN had the lowest performance across all metrics (accuracy: 60.0%, sensitivity: 50.0%, specificity: 69.2%), confirming its limited generalizability on unseen data. These results indicate that Neural Network and Random Forest are the most reliable models for predicting *E. coli* infection on new data. Notably, Naive Bayes achieved the highest AUC (0.843) on the same validation dataset, but its accuracy at the fixed threshold of 0.5 was slightly lower than Neural Network and Random Forest highlighting the complementary nature of threshold-independent (AUC) and threshold-dependent (accuracy, sensitivity, specificity) performance metrics.

## Discussion

HAIs continue to pose a serious global challenge, contributing significantly to patient morbidity, mortality, and increased healthcare expenditure^21,22^. Among the array of pathogens implicated in HAIs, *E. coli* remains a leading cause, particularly in UTIs, bloodstream infections, and surgical site infections. In this study, utilized a no-code ML framework (Orange) for individualized risk prediction of *E.* coli infection across different hospital units. The surveillance study at our institution underscores the predominance of *E. coli* among nosocomial pathogens, with 47.3% (142/300) of bacterial isolates identified as *E. coli* in the training data. This figure is notably higher than those for *Klebsiella pneumoniae* (13%, 39/300), *Candida* spp. (8.7%, 26/300), *Pseudomonas aeruginosa* (2.3%, 7/300), and *Enterobacter* spp. (0.7%, 2/300), highlighting the substantial clinical burden associated with *E. coli* infections. in the clinical setting. These findings are consistent with previous epidemiological reports emphasizing the dominance of *E. coli* in HAIs^1–3^.

In training samples, the majority of *E. coli* isolates were recovered from urine specimens (32.3%), reaffirming its well-established role as the predominant uropathogens in HAIs. Its high isolation rate can be attributed to multiple factors. Firstly, *E. coli* is a common member of the normal gut microbiota, which increases the likelihood of cross-contamination, especially in catheterized or immobilized patients. Secondly, the organism’s ability to persist on inanimate hospital surfaces and medical devices enhances its capacity to cause opportunistic infections^1^. Thirdly, *E. coli* possesses diverse virulence factors, including adhesins, siderophores, and toxins, which facilitate colonization and infection of the urinary tract and other anatomical sites^3^. Another concerning aspect is the increasing prevalence of multidrug-resistant *E. coli*, particularly strains that produce ESBLs. These strains compromise the efficacy of commonly used antibiotics such as penicillin and cephalosporins, necessitating the use of last-resort treatments like carbapenems^4^. This underscores the importance of routine antimicrobial susceptibility testing and the implementation of robust antimicrobial stewardship programs. The data also highlight a substantial percentage (28%) of specimens yielding no growth, which could be due to prior antibiotic administration, improper sampling techniques, or fastidious organisms that were not culturable under standard laboratory conditions. Moreover, the relatively lower incidence of pathogens such as *K. pneumoniae*, *P. aeruginosa*, and *Enterobacter* spp., while still clinically relevant, suggests that tailored infection control strategies should prioritize the most prevalent organisms, notably *E. coli*.

The higher prevalence of *E. coli* among female patients in training data is consistent with well-established epidemiological data. Of the 142 *E. coli*-positive specimens, 82 (57.7%) were from female patients and 60 (42.3%) were from male patients, reflecting a significantly higher burden among females. Several anatomical, physiological, and clinical factors contribute to this observation. The anatomical configuration of the female genitourinary tract facilitates easier ascent of uropathogens from the perineum to the bladder. Additionally, the proximity of the urethral meatus to the anus in females increases the risk of fecal contamination, particularly with *E. coli*, a predominant member of the intestinal microbiota. Hormonal changes may influence vaginal pH, epithelial cell adherence, and local immune responses, potentially increasing susceptibility to colonization and infection. In hospitalized patients, females especially elderly women are more likely to undergo urinary catheterization, a major risk factor for catheter-associated urinary tract infections (CAUTIs), which are commonly caused by *E. coli*. These anatomical, physiological, and clinical factors collectively explain the observed gender disparity in *E. coli* prevalence in our study population^23–27^. The increased susceptibility to *E. coli* infections with advancing age is driven by three interconnected mechanisms: immunosenescence, inflammaging and age-related tissue changes within the urinary tract, particularly the accumulation of advanced glycation end products (AGEs) that enhance bacterial adherence. These immunological and tissue-level vulnerabilities are compounded by clinical factors common in older adults, including multimorbidity, polypharmacy, immobility, urinary retention, malnutrition, and frequent urinary catheterization^28–31^.

From a computational perspective, the integration of ML into clinical workflows provides a powerful tool for analyzing complex and heterogeneous datasets. Recent studies have demonstrated the effectiveness of ML in improving diagnostic accuracy and supporting clinical decision-making^32,33^. In this study, Orange Data Mining software was used as a no-code platform to implement a complete analytical pipeline, enabling data preprocessing, visualization, and predictive modeling without requiring programming expertise^16,34,35^. This aligns with recent advances in AutoML, which aim to simplify model development while maintaining high predictive performance^14^.

In this study, hierarchical clustering widget revealed distinct grouping patterns among hospital departments based on *E. coli* prevelance. While, the Box Plot widget was employed to evaluate the distribution of microbial prevalence across departments. Orange automatically performed a Chi-square test (χ² = 547.45, df = 40, *p* < 0.001) to assess the statistical significance of inter-departmental differences, with results displayed directly on the plot^34^. confirmed statistically significant differences in microbial prevalence across departments. This finding is in agreement with^27^, who emphasized that infection patterns vary substantially by department due to differences in patient demographics, invasive procedures, and antimicrobial exposure. The distinct profiles observed for Urology and Burn Unit both showing high *E. coli* prevalence (88% and 96.2%, respectively) are consistent with the known epidemiology of *E. coli* as the predominant pathogen in catheter-associated UTIs and wound infections. However, our finding of high *E. coli* prevalence in the Burn Unit (96.2%) is notably higher than the 50–70% range reported by Church^36^ in burn patients, potentially reflecting a localized outbreak or differences in infection control practices. These findings are consistent with the broader literature on hospital-acquired infections, which emphasizes the need for tailored antimicrobial stewardship programs based on local epidemiological data. The high prevalence of *E. coli* in Urology and Burn Unit warrants further investigation into infection prevention measures, particularly regarding urinary catheter use and wound care protocols.

The feature ranking analysis identified sample type, hospital department, and diagnosis as the most influential predictors of *E. coli* infection in the training dataset. The use of multiple feature selection techniques (Information Gain, Gain Ratio, Gini, Chi-square, ReliefF, FCBF) ensured robust feature selection and consistent identification of key predictors^37^. From a clinical perspective, the predominance of Diagnosis is expected, as *E. coli* is the leading cause of UTIs and bacteremia^24^. The importance of Department reflects the higher risk in urology and intensive care units, where urinary catheter uses and invasive procedures are more common^38^. Sample Type ranked third, with urine samples being the most informative, consistent with the high prevalence of *E. coli* in UTIs^24,39^. For infection control practice, these findings enable targeted prevention strategies. Infection control teams can focus surveillance and preventive interventions on high-risk departments (urology and ICU), implement catheter-associated UTI prevention bundles, and prioritize urine culture surveillance in these settings^38,40^. For clinical decision-making, physicians can use these predictors to guide empiric antibiotic therapy. When a patient from the urology department presents with suspected infection and a urine sample is submitted, early coverage for *E. coli* should be considered before culture results are available^24,41^. This may reduce time to appropriate therapy and improve patient outcomes.

On 10-fold stratified cross-validation using training dataset, Random Forest achieved the highest AUC of 0.952, followed by Neural Network (0.941) and Naive Bayes (0.940). These findings are consistent with previous studies demonstrating the effectiveness of ensemble and neural network models in clinical prediction tasks^14,37,42,43^. However, training data performance alone can be misleading. When evaluated on an independent validation dataset (*n* = 100), Random Forest dropped to AUC 0.770, while Naive Bayes showed more stable performance (0.940 to 0.843) and achieved the highest AUC (0.843, 95% CI: 0.762–0.924) on internal validation dataset. This pattern is consistent with mild overfitting in the more complex models. Overfitting is a well-recognized challenge in ML, particularly when sample sizes are limited (*n* < 500) and datasets contain complex feature interactions^44,45^. Our findings suggest that for limited sample sizes (*n* = 300), simpler models such as Naive Bayes may generalize better than complex ensemble methods. This is supported by previous empirical studies showing that Naive Bayes reaches optimal performance rapidly and is less prone to overfitting with small training sets^46^. The validation results reflect the predictive potential of the models on unseen data, but do not represent actual clinical prediction. Further prospective evaluation is needed before these models can be used in real-time decision-making.

Several methodological limitations should be considered when interpreting our findings. First, while we report 95% CIs for AUC in the Table 3 caption, calibration analysis was not performed because our internal validation sample size (*n* = 100) is too small for reliable calibration assessment. According to recent methodological guidance^47^, stable calibration requires substantially larger samples (approximately 3,000–12,000 patients). With only 100 samples, calibration estimates would be unreliable and potentially misleading. Therefore, calibration assessment was not pursued. Future studies with larger, multi-center cohorts should evaluate the calibration of our models before clinical implementation.

The confusion matrix analysis provided a detailed assessment of each model’s classification performance on both training and internal validation datasets. On the validation dataset (*n* = 100), Neural Network and Random Forest achieved the highest accuracy (76.0% for both), with Neural Network demonstrating the best specificity (80.8%) and Random Forest showing the highest sensitivity (75.0%). These findings are consistent with previous studies reporting strong predictive performance of ensemble and neural network models for infection prediction tasks. For example, Zhang et al.^48^. developed a Random Forest model to distinguish *E. coli* from other uropathogens using routine clinical data, achieving moderate discrimination (AUC = 0.66) on a held-out test set, with sensitivity and specificity comparable to our findings. Similarly, ML models for antimicrobial resistance prediction in *E. coli* have reported accuracy and AUC values ranging from 0.79 to 0.98 across different classifiers and datasets^48^.

Notably, Naive Bayes showed the smallest performance drop between training and validation (85.3% to 75.0%, a drop of 10.3%), compared to Neural Network (90.3% to 76.0%, a drop of 14.3%) and Random Forest (89.7% to 76.0%, a drop of 13.7%). This finding suggests that simpler models may offer more stable generalizability when working with limited sample sizes. A study evaluating Lyme disease incidence prediction reported comparable performance patterns, where Naive Bayes achieved an AUC of 0.834, while Random Forest achieved 0.895 and Neural Network 0.909, demonstrating the relative stability of simpler models across different prediction tasks. The challenge of generalizability with limited datasets is well-documented in the literature; models trained on small, imbalanced medical datasets often show significant performance drops when applied to new data^49,50^.

While Naive Bayes achieved the highest AUC on validation (0.843), its accuracy at the fixed threshold of 0.5 was slightly lower than that of Neural Network and Random Forest. This highlights the complementary nature of threshold-independent (AUC) and threshold-dependent (accuracy, sensitivity, specificity) metrics in providing a comprehensive evaluation of model performance^51^. In contrast, kNN showed the lowest performance across all validation metrics (accuracy: 60.0%, sensitivity: 50.0%, specificity: 69.2%), confirming its limited generalizability for this prediction task. Other studies have similarly reported lower performance for kNN in clinical classification tasks, with accuracy ranging from 61% to 70% in comparable settings.

The observed performance drop from training to validation (10–14% across models) reflects the expected generalization gap in ML and underscores the importance of internal validation for assessing real-world applicability^49,50^. These findings support the potential of ML-based approaches for early *E. coli* infection prediction, while also highlighting the need for larger, multi-center cohorts to improve model stability and generalizability.

While our findings demonstrate the potential of ML for predicting *E. coli* infection using routine clinical data, several steps remain before clinical application. First, internal validation in larger, multi-center prospective cohorts is required to confirm generalizability. Second, calibration assessment should be performed in sufficiently large samples to ensure reliable probability estimates. Third, prospective evaluation in real-world clinical settings is needed to assess the model’s impact on patient outcomes and antibiotic stewardship. Therefore, the ML should be considered a preliminary tool pending further validation, not a replacement for clinical judgment.

## Conclusion

In conclusion, the findings of this surveillance study emphasize the dominant role of *E. coli* in HAIs, particularly in urinary tract infections, within our clinical setting. The results further demonstrate that ML approaches can support prediction of *E. coli* infection using routinely collected clinical and demographic data. The observed variability in microbial distribution across hospital departments underscores the necessity of tailored, department-specific infection control strategies rather than generalized hospital-wide approaches. In addition, the identification of key predictive factors, such as sample type and department, provides actionable insights for improving infection prevention and management. Among the evaluated classifiers, the Naive Bayes model showed the most stable performance across training and validation datasets, with the smallest performance drop (85.3% to 75.0%) and the highest validation AUC (0.843). These findings suggest that ML-based approaches can support clinical decision-making by identifying high-risk patients before culture results are available, potentially reducing diagnostic delays and supporting antimicrobial stewardship efforts. The use of an accessible, no-code platform (Orange) further highlights the feasibility of integrating predictive analytics into routine hospital workflows, even in settings with limited programming expertise.

However, the observed performance drop from training to validation (10–14% across models) underscores the importance of larger, more diverse datasets to improve model robustness and generalizability. While the internal validation cohort (*n* = 100) provided a realistic assessment of model performance on unseen data, the modest sample size remains a limitation. Future work should focus on training ML models on larger, multi-center cohorts with extended clinical variables (e.g., antibiotic exposure, catheter use, comorbidities) and prospective validation to confirm real-world applicability. Until such validation is completed, the current model should be viewed as a proof-of-concept rather than a ready-to-deploy clinical tool. Nonetheless, the findings reinforce the growing role of ML in infection surveillance and support the continued exploration of data-driven approaches to improve patient outcomes and infection control practices.

## Materials and methods

### Samples collection

A total of 400 samples were collected from various units (NICU, PICU, ICU, burning unit, surgical unit, ENT, and ophthalmic unit) of Zagazig University Hospital in Sharkia, Egypt. Of these, 300 samples were collected between July 2024 and February 2025 as training dataset, and an additional 100 samples were collected during May 2026 as internal validation dataset. Sample types included urine, blood, pus, body fluids, sputum, and wound swabs (see Supplementary Table S1-S8). The samples were collected using sterile suitable containers and the transport swabs were inoculated in normal sterile saline. After being cultured on blood agar and MacConkey plates. These plates underwent an overnight aerobic incubation at 37^◦^C before being examined for bacterial growth.

### Morphological and biochemical tests for identification of selected isolate

**Fig. 8:**
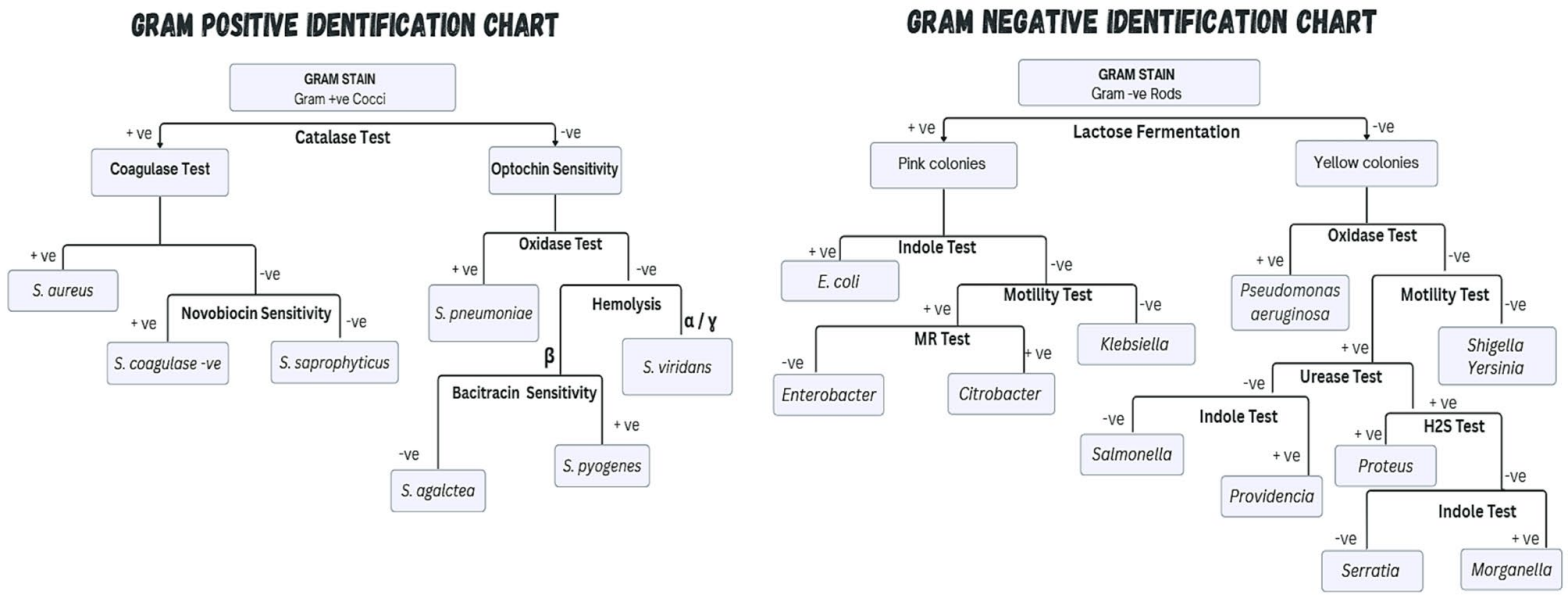
Flow chart for Gram positive and negative bacteria identification.

A colony was picked from each plate with suspected growth and streaked onto plates containing nutrient agar (CM0003, Oxoid, UK) and incubated for 16–18 h at 37 °C for purification. The isolates were then microscopically examined by gram staining to differentiate the isolates based on cell wall composition^52^. Then, all purified isolates were examined by biochemical tests (Fig. 8) according to Bergey’s manual of determinative bacteriology^53^. All results then confirmed by the VITEK 2 automated system (bioMérieux, France).

### Data analytics and machine learning modeling

ML analyses were performed using Orange Data Mining (Version 3.40), an open-source visual programming (no-code) tool. The analytical workflow was designed to transition from basic statistical characterization to advanced multivariate analysis and ML based feature importance^54^.

#### Preprocessing and data loading

**Fig. 9:**
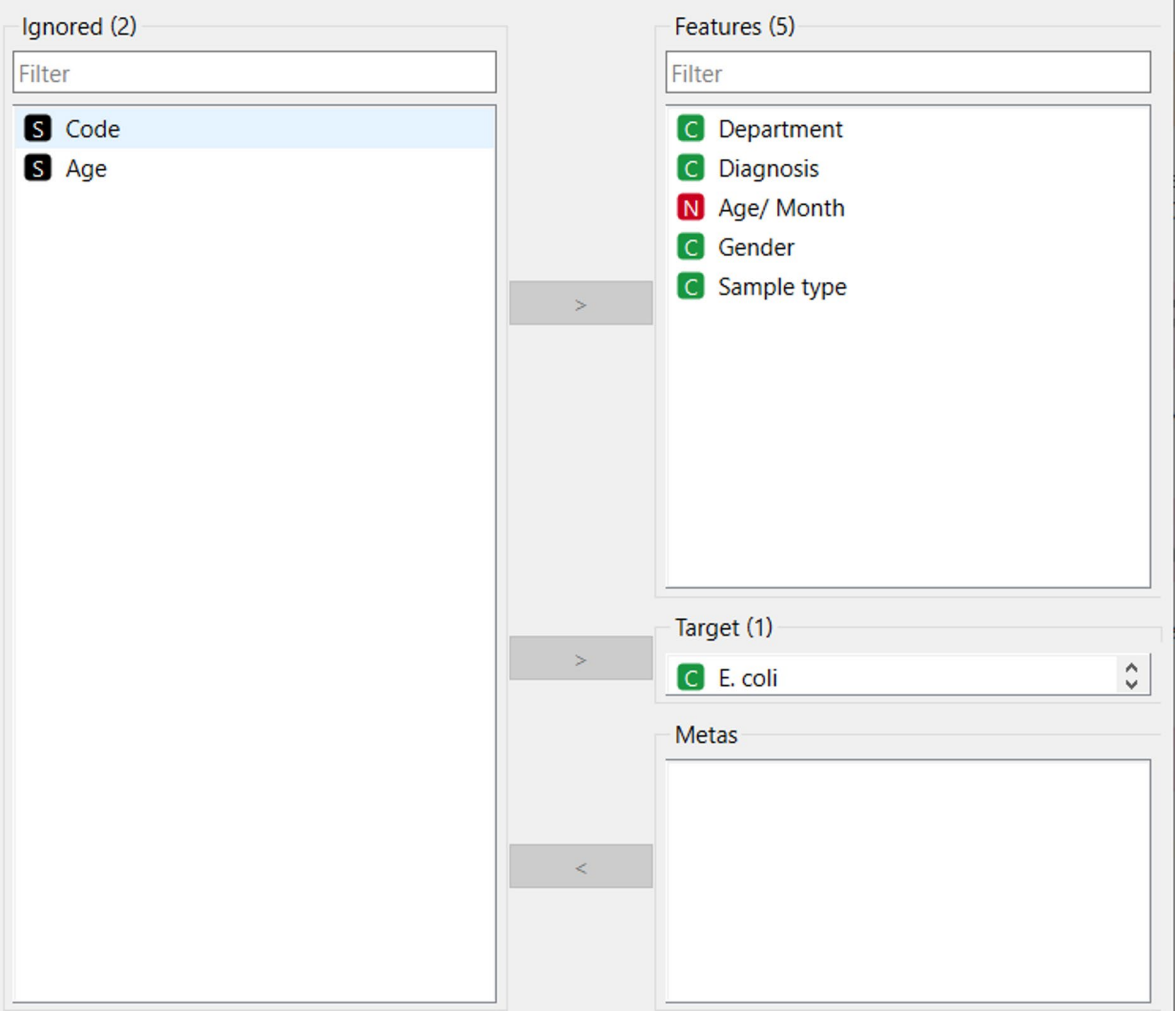
Data structure and variable roles as shown in Orange Data Mining. All input variables (Department, Diagnosis, Age/Month, Gender, Sample type), their types, and the target variable (*E. coli*). Variables marked as ‘ignored’ (Code, Age) were excluded from the analysis.

Before model development, the dataset was prepared in Excel sheet through several preprocessing steps to ensure data quality and prevent data leakage, special attention was given to handling textual entries and spacing inconsistencies. Five predictor variables were included in the analysis: Department, Diagnosis, Age/Month, Gender, and Sample Type. The target variable was *E. coli* infection status: patients with a positive culture result for *E. coli* from any clinical specimen (urine, blood, body fluid, pus, or sputum) were coded as 1 (positive), while all other patients, including those with no growth or growth of other microorganisms, were coded as 0 (negative). This binary outcome was used for all supervised learning tasks. The age variable was uniformly recorded in months across all samples to achieve higher precision, especially for infants and pediatric patients. No missing values were present in any of the included variables for both the training dataset (*n* = 300) and the internal validation dataset (*n* = 100), and all records were complete. No extreme outliers were detected in the Age variable; therefore, no imputation or outlier capping was necessary. To prevent data leakage in model evaluation, the culture result column was excluded from the input features, besides, the diagnosis variable was reviewed; it contains the initial clinical diagnosis recorded prior to microbiological culture results, and includes only clinical syndromes (e.g., urinary tract infection, bacteremia, anemia, urine retention) with no specific organism names (e.g., no *E. coli*, *Klebsiella*). Therefore, it does not constitute data leakage (Fig. 9).

**Fig. 10:**
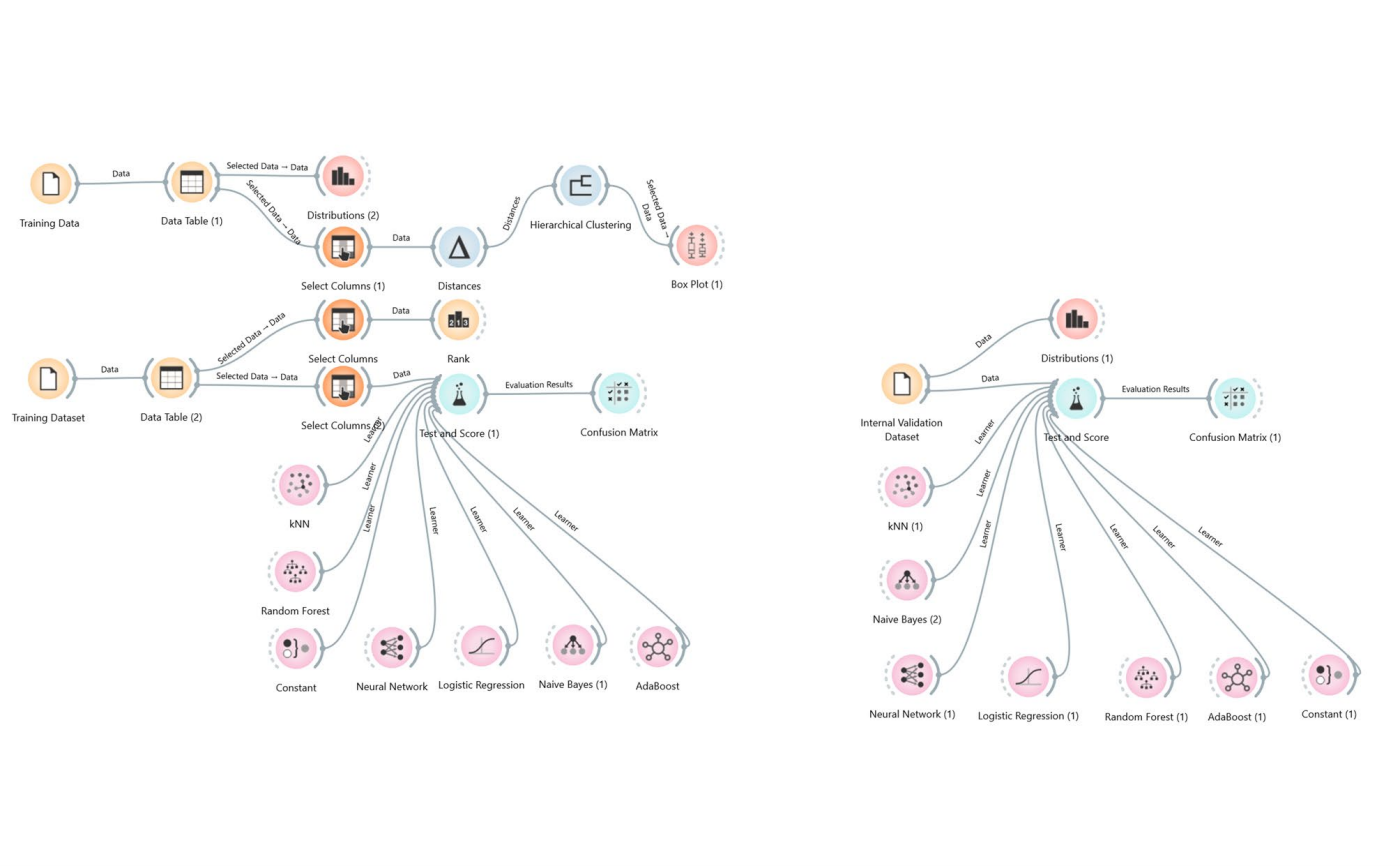
Workflow of preprocessing, model development, evaluation, and explainability using Orange Data Mining.

The workflow began with loading the training dataset worksheet after preprocessing step and the internal validation dataset (*n* = 100) using separate Data widgets as shown in Fig. 10. The training dataset was used for visualization, ranking features and model development, while the internal validation dataset was kept completely separate for independent model evaluation.

#### Visualization and graphical analysis

Orange operates on a visual programming paradigm, where analytical workflows are constructed by linking functional components (widgets). Each widget performs a specific computational task, such as data transformation, model training, or visualization, and passes its output to subsequent components. This modular architecture enhances reproducibility, transparency, and ease of use, particularly for interdisciplinary applications involving clinical and computational domains.

To explore patterns in the data and support model interpretability, we employed three main visualization widgets within the Orange platform. The Distribution widget was used to examine the relationship between department and sample type, revealing which specimen types were most frequently collected in each department (Fig. 1). The Box Plot widget was used to visualize the distribution of all bacterial isolates of the training data only across different departments, allowing comparison of microbial prevalence patterns. The Hierarchical Clustering (HCA) widget was applied to the training dataset to group departments based on *E. coli* prevalence, with the dendrogram color-coded according to the proportion of *E. coli*-positive cases. The dendrogram was generated using the Average linkage method and was displayed without pruning to preserve the full hierarchical structure of the data.

#### Model interpretability and explainable artificial intelligence (XAI)

To improve transparency and clinical interpretability, model explanation approaches were incorporated into the ML workflow. Although predictive performance is an important criterion, understanding the factors influencing model decisions is essential for the clinical adoption of artificial intelligence systems^16,18^. In this study, model interpretability was addressed through feature ranking and importance analysis to identify the variables contributing most significantly to *E. coli* infection prediction. Multiple feature evaluation methods, including Information Gain, Gain Ratio, Gini Index, Chi-square, ReliefF, and FCBF, were applied to assess the contribution of each variable across the dataset (Fig. 5).

These approaches provided global interpretability by identifying the most influential predictors, such as specimen type, hospital department, and diagnosis, which contributed to the overall prediction process. In addition, visualization-based analysis facilitated the understanding of relationships between clinical variables and model outcomes, supporting interpretation by both computational and clinical researchers. By integrating feature-based interpretation with ML prediction, the proposed framework follows human-centered AI principles and promotes the development of transparent, explainable, and clinically applicable predictive models.

#### Model development and evaluation

Multiple supervised ML models were trained and evaluated, including Random Forest, AdaBoost, Neural Networks, Logistic Regression, Naive Bayes, and kNN on the training data (Fig. 10). These models were selected to represent both ensemble-based and probabilistic learning approaches, enabling comparative performance analysis. Model evaluation was conducted using a comprehensive set of performance metrics, including accuracy, sensitivity, specificity, precision, recall, F1-score, Receiver Operating Characteristic-AUC (ROC-AUC), Precision-Recall- AUC (PR-AUC), and Brier score. This multi-metric evaluation strategy provides a robust assessment of classification performance, discrimination ability, and probability calibration. For all classifiers, the default classification threshold of 0.5 was used to convert predicted probabilities into binary class labels. This is the standard default in scikit-learn, the core library underlying Orange Data Mining^20^. Specifically, a sample was classified as positive (*E. coli* infection = 1) if the predicted probability was ≥ 0.5, and negative (0) otherwise. This threshold was applied to calculate all threshold-dependent performance metrics (accuracy, F1, precision, recall, and MCC).

To evaluate generalizability, internal validation was performed using an independent dataset of 100 samples collected during May 2026. These samples were never used in model training or hyperparameter tuning. All seven classifiers (kNN, Logistic Regression, Neural Network, Random Forest, Naive Bayes, AdaBoost, and Constant) were applied to this dataset without any re-training. Bootstrap resampling with 1,000 iterations was used to calculate 95% CIs for all performance metrics, including AUC, accuracy, sensitivity, and specificity^55^. The bias-corrected and accelerated (BCa) method was applied. All statistical analyses were performed using open-source software.

To evaluate and visualize the performance of the trained classifiers, we used the Confusion Matrix widget in Orange. After training the models (kNN, Logistic Regression, Neural Network, Random Forest, Naive Bayes, AdaBoost, and Constant) and evaluating them on both the training data (Fig. 6) and internal validation datasets (Fig. 7) using the Test & Score widget, the resulting evaluation results were passed to the Confusion Matrix widget. For each model, this generated a table displaying the counts of true positives (TP), true negatives (TN), false positives (FP), and false negatives (FN). These matrices were used to calculate key performance metrics, including accuracy, sensitivity (recall), and specificity for each model on both datasets. Sensitivity (recall) was defined as TP/(TP + FN), specificity as TN/(TN + FP), PPV as TP/(TP + FP), and NPV as TN/(TN + FN). The visual representation of the confusion matrices aided in comparing model performance and identifying which classifiers made fewer errors in predicting *E. coli* infection.

## Supplementary Information

Below is the link to the electronic supplementary material.

Supplementary Material 1

Supplementary Material 2

Supplementary Material 3

## Data Availability

The datasets utilized and/or examined in the present study can be obtained by contacting the corresponding author and its Supplementary materials files.

## Code availability

All primary analyses were performed using Orange Data Mining version 3.40 (https://orangedatamining.com), an open-source visual programming platform. No custom code was written for the main prediction workflow. The complete Orange workflow file (.ows) containing all analysis steps (data preprocessing, feature selection, model training, evaluation, and bootstrap resampling for confidence intervals) is provided as Supplementary File 2. The workflow file is sufficient to reproduce all results reported in this manuscript. The 95% confidence intervals for the Area Under the Curve (AUC) were calculated using a non-parametric bootstrap resampling method implemented in Python version 3.9 with the scikit-learn library version 1.1 (https://scikit-learn.org/). The implementation was adapted from a standard, community-validated method (Stack Overflow, 2013) under the CC BY-SA 4.0 license. The analysis was performed with 1,000 bootstrap iterations. For each iteration, the dataset was resampled with replacement, and the AUC was recomputed. The 2.5th and 97.5th percentiles of the resulting distribution defined the 95% confidence interval. The complete Python script is provided as Supplementary Code 1. All open-source software used (Orange, Python, scikit-learn) are freely available online. The Supplementary files are also available from the corresponding author upon reasonable request.
