# Supplementary material for: Machine learning-based prediction of E. coli infection in hospitalized patients using a no-code analytical framework
Source: Sci Rep. 2026 Jul 4;16:20563. doi: 10.1038/s41598-026-59795-y (PMC13333015; doi:10.1038/s41598-026-59795-y)
Supplement: Supplementary file 1 — Supplementary Material 1 [file 41598_2026_59795_MOESM1_ESM.docx]

**Supplementary Table S1:** **Isolated Microorganisms from Different Samples from Urology Unit.**

| **S.NO** | **Code** | **Age** | **Gender** | **Sample type** | **MOs** | ***E. coli*** |
| --- | --- | --- | --- | --- | --- | --- |
| **1** | UTI 1 | 87 Y | Female | Pus | *Klebsiella pneumoniae* | *0* |
| **2** | UTI 4 | 63 Y | Female | Body fluid | *E. coli* | *1* |
| **3** | UTI 7 | 38 Y | Female | Urine | *E. coli* | *1* |
| **4** | UTI 9 | 73 Y | Male | Urine | *E. coli* | *1* |
| **5** | UTI 10 | 80 Y | Male | Urine | *E. coli* | *1* |
| **6** | UR 17 | 63 Y | Male | Body fluid | *E. coli* | *1* |
| **7** | UTI 26 | 61 Y | Male | Pus | *E. coli* | *1* |
| **8** | UTI 27 | 38 Y | Female | Urine | *E. coli* | *1* |
| **9** | UTI 39 | 56 Y | Female | Body fluid | *E. coli* | *1* |
| **10** | UTI 41 | 40 Y | Male | Urine | *E. coli* | *1* |
| **11** | UIRR 43 | 32 Y | Female | Urine | *E. coli* | *1* |
| **12** | UTI 44 | 47 Y | Female | Urine | *E. coli* | *1* |
| **13** | UTI 49 | 63 Y | Female | Blood | *E. coli* | *1* |
| **14** | UTI 51 | 36 Y | Female | Urine | *E. coli* | *1* |
| **15** | UTI 55 | 25 Y | Female | Urine | *E. coli* | *1* |
| **16** | UTI 63 | 71 Y | Female | Urine | *E. coli* | *1* |
| **17** | GENI 64 | 74 Y | Male | Urine | *Klebsiella pneumoniae* | *0* |
| **18** | HEMU 65 | 60 Y | Male | Urine Catheter | *E. coli* | *1* |
| **19** | GEN.PNEU 67 | 38 Y | Female | Sputum | *Klebsiella pneumoniae* | *0* |
| **20** | UIRR 69 | 63 Y | Male | Urine | *E. coli* | *1* |
| **21** | UTI 70 | 52 Y | Female | Urine | *E. coli* | *1* |
| **22** | UTI 71 | 52 Y | Female | Urine | *E. coli* | *1* |
| **23** | HEMU 72 | 60 Y | Male | Urine Catheter | *Candida albicans* | *0* |
| **24** | GEN.PNEU 73 | 48 Y | Female | Sputum | *Klebsiella pneumoniae* | *0* |
| **25** | UIRR 78 | 63 Y | Male | Urine | *E. coli* | *1* |
| **26** | UTI 83 | 71 Y | Female | Urine | *E. coli* | *1* |
| **27** | UIRR 89 | 63 Y | Male | Urine | *E. coli* | *1* |
| **28** | UTI 91 | 56 Y | Female | Urine | *E. coli* | *1* |
| **29** | UTI 97 | 53 Y | Female | Urine | *E. coli* | *1* |
| **30** | UIRR 101 | 52 Y | Female | Urine | *E. coli* | *1* |
| **31** | UR 105 | 47 Y | Male | Urine | *E. coli* | *1* |
| **32** | UR 107 | 63 Y | Male | Body fluid | *E. coli* | *1* |
| **33** | UTI 111 | 56 Y | Female | Urine | *E. coli* | *1* |
| **34** | UIRR 115 | 51 Y | Female | Urine | *E. coli* | *1* |
| **35** | UTI 117 | 47 Y | Female | Urine | *E. coli* | *1* |

**^UTI: Urinary Tract Infection, UR: Urine Retention, HEMU: Hematuria, GENI: Genital Infection, PNEU: Pneumonia, UIRR: Urination Irregular.^**

**Supplementary Table S1 (Continue):** **Isolated Microorganisms from Different Samples from Urology Unit.**

| **S.NO** | **Code** | **Age** | **Gender** | **Sample type** | **MOs** | ***E. coli*** |
| --- | --- | --- | --- | --- | --- | --- |
| **36** | UTI 120 | 36 Y | Female | Urine | *E. coli* | *1* |
| **37** | UTI 121 | 69 Y | Female | Pus | *Klebsiella pneumoniae* | *0* |
| **38** | UTI 124 | 62 Y | Female | Body fluid | *E. coli* | *1* |
| **39** | UTI 127 | 53 Y | Female | Urine | *E. coli* | *1* |
| **40** | UTI 129 | 36 Y | Female | Urine | *E. coli* | *1* |
| **41** | UTI 130 | 74 Y | Male | Urine | *E. coli* | *1* |
| **42** | UTI 131 | 87 Y | Female | Pus | *Klebsiella pneumoniae* | *0* |
| **43** | UTI 134 | 52 Y | Male | Body fluid | *E. coli* | *1* |
| **44** | UTI 137 | 38 Y | Female | Urine | *E. coli* | *1* |
| **45** | UTI 138 | 36 Y | Female | Urine | *E. coli* | *1* |
| **46** | UTI 139 | 73 Y | Male | Urine | *E. coli* | *1* |
| **47** | UTI 140 | 80 Y | Male | Urine | *E. coli* | *1* |
| **48** | UTI 141 | 56 Y | Female | Urine | *E. coli* | *1* |
| **49** | UTI 145 | 45 Y | Female | Urine | *E. coli* | *1* |
| **50** | UTI 147 | 52 Y | Male | Urine | *Candida albicans* | *0* |
| **51** | UTI 149 | 60 Y | Female | Urine | *E. coli* | *1* |
| **52** | UTI 151 | 77 Y | Male | Urine | *E. coli* | *1* |
| **53** | UTI 157 | 52 Y | Female | Urine | *E. coli* | *1* |
| **54** | UTI 160 | 36 Y | Female | Urine | *E. coli* | *1* |
| **55** | UTI 161 | 56 Y | Female | Urine | *E. coli* | *1* |
| **56** | UTI 170 | 40 Y | Male | Urine | *E. coli* | *1* |
| **57** | UTI 171 | 63 Y | Female | Blood | *E. coli* | *1* |
| **58** | UTI 175 | 25 Y | Female | Urine | *E. coli* | *1* |
| **59** | HEMU 177 | 32 Y | Male | Urine | *E. coli* | *1* |
| **60** | UTI 184 | 63 Y | Female | Body fluid | *E. coli* | *1* |
| **61** | GENI 186 | 32 Y | Male | Urine | No growth | *0* |
| **62** | UTI 187 | 38 Y | Female | Urine | *E. coli* | *1* |
| **63** | UTI 190 | 73 Y | Male | Urine | *E. coli* | *1* |
| **64** | UTI 193 | 68 Y | Female | Urine | *E. coli* | *1* |
| **65** | UIRR 199 | 65 Y | Male | Urine | *E. coli* | *1* |
| **66** | UIRR 201 | 49 Y | Female | Urine | *E. coli* | *1* |
| **67** | UTI 202 | 56 Y | Male | Urine | *E. coli* | *1* |
| **68** | UR 203 | 53 Y | Female | Urine | *E. coli* | *1* |
| **69** | UIRR 213 | 47 Y | Female | Urine | *E. coli* | *1* |
| **70** | UIRR 215 | 32 Y | Female | Urine | *E. coli* | *1* |

**^UTI: Urinary Tract Infection, UR: Urine Retention, HEMU: Hematuria, GENI: Genital Infection, PNEU: Pneumonia, UIRR: Urination Irregular.^**

**Supplementary Table S1 (Continue):** **Isolated Microorganisms from Different Samples from Urology Unit.**

| **S.NO** | **Code** | **Age** | **Gender** | **Sample type** | **MOs** | ***E. coli*** |
| --- | --- | --- | --- | --- | --- | --- |
| **71** | UR 217 | 63 Y | Male | Body fluid | *E. coli* | *1* |
| **72** | UR 222 | 52 Y | Male | Urine | *E. coli* | *1* |
| **73** | UTI 223 | 52 Y | Male | Body fluid | *E. coli* | *1* |
| **74** | UR 227 | 60 Y | Male | Body fluid | *E. coli* | *1* |
| **75** | UIRR 231 | 52 Y | Female | Urine | *E. coli* | *1* |
| **76** | UIRR 233 | 47 Y | Female | Urine | *E. coli* | *1* |
| **77** | UR 237 | 63 Y | Male | Body fluid | *E. coli* | *1* |
| **78** | UTI 244 | 62 Y | Female | Body fluid | No growth | *0* |
| **79** | UTI 247 | 64 Y | Female | Urine | *E. coli* | *1* |
| **80** | UIRR 248 | 56 Y | Male | Urine | *Candida albicans* | *0* |
| **81** | UTI 250 | 76 Y | Male | Urine | *E. coli* | *1* |
| **82** | UIRR 251 | 50 Y | Male | Urine | *E. coli* | *1* |
| **83** | UR 253 | 47 Y | Female | Urine | *E. coli* | *1* |
| **84** | UIRR 261 | 52 Y | Female | Urine | *E. coli* | *1* |
| **85** | UR 265 | 47 Y | Male | Urine | *E. coli* | *1* |
| **86** | UR 267 | 63 Y | Male | Body fluid | *E. coli* | *1* |
| **87** | UTI 271 | 36 Y | Female | Urine | *E. coli* | *1* |
| **88** | UTI 277 | 38 Y | Female | Urine | *E. coli* | *1* |
| **89** | UTI 281 | 45 Y | Female | Urine | *E. coli* | *1* |
| **90** | UTI 287 | 52 Y | Female | Urine | *E. coli* | *1* |
| **91** | UTI 293 | 68 Y | Female | Urine | *E. coli* | *1* |
| **92** | UIRR 299 | 65 Y | Male | Urine | *E. coli* | *1* |

**^UTI: Urinary Tract Infection, UR: Urine Retention, HEMU: Hematuria, GENI: Genital Infection, PNEU: Pneumonia, UIRR: Urination Irregular.^**

**Supplementary Table S2:** **Isolated Microorganisms from Different Samples from Neonates Intensive Care Unit (NICU).**

| **S.NO** | **Code** | **Age** | **Gender** | **Sample type** | **MOs** | ***E. coli*** |
| --- | --- | --- | --- | --- | --- | --- |
|  | BCEM 3 | 1 D | Male | Blood | *Klebsiella pneumoniae* | *0* |
|  | B.HEM 15 | 2 M | Female | Blood | No growth | *0* |
|  | B.HEM 16 | 3 D | Male | Blood | No growth | *0* |
|  | BCEM 19 | 2 D | Male | Blood | No growth | *0* |
|  | B.HEM 20 | 1 M | Female | Blood | No growth | *0* |
|  | BCEM 21 | 1 D | Male | Blood | No growth | *0* |
|  | BCEM 45 | 1 D | Female | Blood | No growth | *0* |
|  | BCEM 46 | 3 M | Male | Blood | No growth | *0* |
|  | PNEU 53 | 15 D | Male | Sputum | *Klebsiella pneumoniae* | *0* |
|  | HEMU 57 | 14 D | Male | Urine | No growth | *0* |
|  | UTI 59 | 14 D | Male | Urine | No growth | *0* |
|  | BCEM 99 | 1 D | Male | Blood | No growth | *0* |
|  | B.HEM 106 | 2 M | Female | Blood | No growth | *0* |
|  | BCEM 109 | 1 D | Male | Blood | No growth | *0* |
|  | BCEM 119 | 2 D | Male | Blood | No growth | *0* |
|  | BCEM 123 | 1 D | Male | Blood | *Klebsiella pneumoniae* | *0* |
|  | BCEM 159 | 2 D | Male | Blood | No growth | *0* |
|  | B.HEM 166 | 1 M | Male | Blood | No growth | *0* |
|  | BCEM 168 | 2 D | Male | Blood | No growth | *0* |
|  | UTI 179 | 1 D | Male | Urine | No growth | *0* |
|  | B.HEM 196 | 1 M | Female | Blood | No growth | *0* |
|  | B.HEM 210 | 1 M | Female | Blood | No growth | *0* |
|  | B.HEM 216 | 1 D | Male | Blood | No growth | *0* |
|  | B.HEM 220 | 1 M | Female | Blood | No growth | *0* |
|  | B.HEM 225 | 1 M | Female | Blood | No growth | *0* |
|  | BCEM 229 | 1 D | Male | Blood | No growth | *0* |
|  | B.HEM 235 | 2 M | Female | Blood | No growth | *0* |
|  | B.HEM 256 | 3 D | Male | Blood | No growth | *0* |
|  | B.HEM 266 | 2 M | Female | Blood | No growth | *0* |
|  | BCEM 269 | 1 D | Male | Blood | *E. coli* | *1* |
|  | B.HEM 276 | 2 M | Female | Blood | No growth | *0* |
|  | BCEM 279 | 2 D | Male | Blood | No growth | *0* |
|  | BCEM 280 | 5 D | Male | Blood | *Klebsiella pneumoniae* | *0* |
|  | B.HEM 296 | 1 M | Female | Blood | No growth | *0* |

**^BCEM: Bacteremia, B.HEM: Blood Hemolysis, PNEU: Pneumonia, UTI: Urinary Tract Infection, HEMU: Hematuria.^**

**Supplementary Table S3:** **Isolated Microorganisms from Different Samples from Pediatric Intensive Care Unit (PICU).**

| **S.NO** | **Code** | **Age** | **Gender** | **Sample type** | **MOs** | ***E. coli*** |
| --- | --- | --- | --- | --- | --- | --- |
|  | GENI 6 | 8 Y | Male | Urine | No growth | *0* |
|  | BCEM 18 | 12 Y | Female | Blood | *E. coli* | *1* |
|  | BCEM 23 | 7 Y | Male | Blood | *E. coli* | *1* |
|  | BCEM 24 | 2 Y | Female | Blood | *E. coli* | *1* |
|  | UTI 56 | 9 Y | Male | Urine | *E. coli* | *1* |
|  | BCEM 58 | 1 Y | Male | Blood | No growth | *0* |
|  | BCEM 68 | 11 Y | Female | Blood | No growth | *0* |
|  | B.HEM 86 | 6 M | Female | Blood | No growth | *0* |
|  | BCEM 88 | 5 Y | Female | Urine | *E. coli* | *1* |
|  | ANM 90 | 12 Y | Female | Blood | No growth | *0* |
|  | B.HEM 96 | 10 M | Male | Blood | No growth | *0* |
|  | BCEM 98 | 10 Y | Female | Urine | *E. coli* | *1* |
|  | GENI 126 | 8 Y | Male | Urine | No growth | *0* |
|  | BCEM 133 | 1 Y | Female | Blood | *Klebsiella pneumoniae* | *0* |
|  | GENI 136 | 12 Y | Male | Urine | No growth | *0* |
|  | B.HEM 156 | 8 M | Male | Blood | No growth | *0* |
|  | BCEM 167 | 5 Y | Female | Urine | *E. coli* | *1* |
|  | UTI 176 | 9 Y | Male | Urine | *E. coli* | *1* |
|  | BCEM 178 | 2 Y | Male | Blood | No growth | *0* |
|  | BCEM 198 | 10 Y | Female | Urine | No growth | *0* |
|  | ANM 200 | 12 Y | Female | Blood | No growth | *0* |
|  | BCEM 208 | 12 Y | Female | Blood | No growth | *0* |
|  | BCEM 218 | 12 Y | Female | Blood | No growth | *0* |
|  | BCEM 228 | 1 Y | Female | Blood | *E. coli* | *1* |
|  | BCEM 238 | 12 Y | Female | Blood | No growth | *0* |
|  | BCEM 273 | 9 Y | Female | Blood | *E. coli* | *1* |
|  | BCEM 278 | 5 Y | Female | Urine | *E. coli* | *1* |
|  | BCEM 298 | 10 Y | Female | Urine | No growth | *0* |
|  | ANM 300 | 12 Y | Female | Blood | No growth | *0* |

**^BCEM: Bacteremia, B.HEM: Blood Hemolysis, UTI: Urinary Tract Infection, GENI: Genital Infection, ANM: Anemia.^**

**Supplementary Table S4:** **Isolated Microorganisms from Different Samples from Intensive Care Unit (ICU).**

| **S.NO** | **Code** | **Age** | **Gender** | **Sample type** | **MOs** | ***E. coli*** |
| --- | --- | --- | --- | --- | --- | --- |
| **1** | PNEU 5 | 81 Y | Male | Blood | No growth | *0* |
| **2** | SEPT 8 | 84 Y | Female | Blood | No growth | *0* |
| **3** | ANM 22 | 69 Y | Male | Blood | No growth | *0* |
| **4** | B.HEM 28 | 85 Y | Male | Urine | No growth | *0* |
| **5** | RESI 29 | 84 Y | Female | Sputum | *Candida albicans* | *0* |
| **6** | BCEM 30 | 63 Y | Male | Blood | *E. coli* | 1 |
| **7** | BCEM 31 | 69 Y | Male | Blood | *E. coli* | 1 |
| **8** | ANM 32 | 76 Y | Female | Blood | No growth | *0* |
| **9** | DYS 36 | 70 Y | Male | Urine | *Enterobacter* | *0* |
| **10** | PUR 40 | 50 Y | Female | Pus | No growth | *0* |
| **11** | B.HEM 47 | 35 Y | Male | Blood | No growth | *0* |
| **12** | SCOP 48 | 62 Y | Male | Sputum | *Klebsiella pneumoniae* | *0* |
| **13** | MYAL 50 | 45 Y | Male | Blood | *Klebsiella pneumoniae* | *0* |
| **14** | HPYR 52 | 63 Y | Female | Sputum | *Klebsiella pneumoniae* | *0* |
| **15** | SCOP 61 | 62 Y | Male | Sputum | *Klebsiella pneumoniae* | *0* |
| **16** | MEN 62 | 75 Y | Male | Blood | *E. coli* | 1 |
| **17** | B.HEM 66 | 62 Y | Male | Body fluid | No growth | *0* |
| **18** | BCTI 75 | 55 Y | Male | Pus | No growth | *0* |
| **19** | BCTI 76 | 35 Y | Male | Pus | *E. coli* | 1 |
| **20** | MEN 77 | 83 Y | Male | Blood | *E. coli* | 1 |
| **21** | BCEM 80 | 51 Y | Male | Blood | *Klebsiella pneumoniae* | *0* |
| **22** | PNEU 81 | 81 Y | Male | Blood | No growth | *0* |
| **23** | B.HEM 82 | 51 Y | Male | Blood | *Klebsiella pneumoniae* | *0* |
| **24** | BCTI 85 | 61 Y | Male | Pus | *E. coli* | 1 |
| **25** | BCEM 92 | 62 Y | Male | Blood | *E. coli* | 1 |
| **26** | BCEM 93 | 59 Y | Female | Blood | *E. coli* | 1 |
| **27** | BCEM 95 | 81 Y | Male | Blood | *E. coli* | 1 |
| **28** | ANM 100 | 52 Y | Male | Blood | *Klebsiella pneumoniae* | *0* |
| **29** | BCEM 108 | 29 Y | Female | Blood | No growth | *0* |
| **30** | B.HEM 110 | 66 Y | Female | Blood | No growth | *0* |
| **31** | BCEM 113 | 59 Y | Female | Blood | *E. coli* | 1 |
| **32** | PNEU 125 | 81 Y | Male | Blood | No growth | *0* |
| **33** | SEPT 128 | 84 Y | Female | Blood | No growth | *0* |

**^PNEU: Pneumonia, SEPT: Septicemia, ANM: Anemia, B.HEM: Blood Hemolysis, RESI: Respiratory Infection, BCEM: Bacteremia, DYS: Dyspnea, PUR: Purulent, SCOP: Sputum Copious, MYAL: Myalgia, HPYR: Hyperpyrexia, PYR: Pyrexia, BCTI: Bacterial infection, MEN: Meningitis.^**

**Supplementary Table S4 (Continue):** **Isolated Microorganisms from Different Samples from Intensive Care Unit (ICU).**

| **S.NO** | **Code** | **Age** | **Gender** | **Sample type** | **MOs** | ***E. coli*** |
| --- | --- | --- | --- | --- | --- | --- |
| **34** | PNEU 135 | 63 Y | Male | Blood | No growth | *0* |
| **35** | BCEM 143 | 60 Y | Female | Blood | *E. coli* | 1 |
| **36** | ANM 150 | 45 Y | Male | Blood | *Klebsiella pneumoniae* | *0* |
| **37** | BCEM 153 | 59 Y | Female | Blood | *E. coli* | *1* |
| **38** | BCEM 155 | 84 Y | Male | Blood | *E. coli* | *1* |
| **39** | BCEM 163 | 59 Y | Female | Blood | *E. coli* | *1* |
| **40** | BCEM 165 | 84 Y | Male | Blood | *E. coli* | *1* |
| **41** | ANM 169 | 45 Y | Male | Blood | *Klebsiella pneumoniae* | *0* |
| **42** | PYR 172 | 58 Y | Female | Sputum | *Klebsiella pneumoniae* | *0* |
| **43** | RESI 173 | 28 Y | Male | Sputum | *Klebsiella pneumoniae* | *0* |
| **44** | RESI 182 | 55 Y | Female | Sputum | *Pseudomonas aeruginosa* | *0* |
| **45** | PNEU 185 | 81 Y | Male | Blood | No growth | *0* |
| **46** | SEPT 188 | 74 Y | Female | Blood | No growth | *0* |
| **47** | BCEM 191 | 60 Y | Male | Blood | *E. coli* | *1* |
| **48** | B.HEM 192 | 50 Y | Male | Blood | *Klebsiella* | *0* |
| **49** | BCTI 195 | 72 Y | Male | Pus | No growth | *0* |
| **50** | B.HEM 205 | 52 Y | Female | Blood | No growth | *0* |
| **51** | B.HEM 206 | 64 Y | Male | Blood | No growth | *0* |
| **52** | BCEM 239 | 64 Y | Male | Blood | *E. coli* | *1* |
| **53** | B.HEM 240 | 63 Y | Male | Blood | No growth | *0* |
| **54** | RESI 242 | 72 Y | Female | Sputum | *Pseudomonas aeruginosa* | *0* |
| **55** | RESI 245 | 65 Y | Female | Sputum | *Pseudomonas aeruginosa* | *0* |
| **56** | BCEM 258 | 25 Y | Female | Blood | *E. coli* | *1* |
| **57** | BCEM 259 | 36 Y | Female | Blood | *E. coli* | *1* |
| **58** | BCEM 268 | 29 Y | Female | Blood | No growth | *0* |
| **59** | B.HEM 270 | 66 Y | Female | Blood | No growth | *0* |
| **60** | BCTI 275 | 55 Y | Male | Pus | No growth | *0* |
| **61** | BCEM 283 | 52 Y | Female | Blood | *E. coli* | *1* |
| **62** | BCEM 285 | 84 Y | Male | Blood | *E. coli* | *1* |
| **63** | BCTI 288 | 25 Y | Female | Urine | No growth | *0* |
| **64** | ANM 290 | 45 Y | Male | Blood | *Klebsiella pneumoniae* | *0* |
| **65** | BCEM 291 | 60 Y | Male | Blood | *E. coli* | *1* |
| **66** | B.HEM 292 | 50 Y | Male | Blood | *Klebsiella pneumoniae* | *0* |
| **67** | BCTI 295 | 72 Y | Male | Pus | No growth | *0* |

**^PNEU: Pneumonia, SEPT: Septicemia, ANM: Anemia, B.HEM: Blood Hemolysis, RESI: Respiratory Infection, BCEM: Bacteremia, DYS: Dyspnea, PUR: Purulent, SCOP: Sputum Copious, MYAL: Myalgia, HPYR: Hyperpyrexia, PYR: Pyrexia, BCTI: Bacterial infection, MEN: Meningitis.^**

**Supplementary Table S5:** **Isolated Microorganisms from Different Samples from Burning Care Unit.**

| **S.NO** | **Code** | **Age** | **Gender** | **Sample type** | **MOs** | ***E. coli*** |
| --- | --- | --- | --- | --- | --- | --- |
|  | BRIN 11 | 50 Y | Female | Urine | *E. coli* | *1* |
|  | BRIN 34 | 30 Y | Female | Urine | *E. coli* | *1* |
|  | BRIN 37 | 45 Y | Female | Urine | *E. coli* | *1* |
|  | BRIN 42 | 55 Y | Male | Urine | *E. coli* | *1* |
|  | BRIN 74 | 63 Y | Female | Urine | *E. coli* | *1* |
|  | BRIN 84 | 52 Y | Male | Urine | *E. coli* | *1* |
|  | BRIN 87 | 38 Y | Female | Urine | *E. coli* | *1* |
|  | BRIN 94 | 49 Y | Female | Urine | *E. coli* | *1* |
|  | BRIN 114 | 50 Y | Female | Urine | *E. coli* | *1* |
|  | BRIN 144 | 50 Y | Female | Urine | *E. coli* | *1* |
|  | BRIN 154 | 50 Y | Female | Urine | *E. coli* | *1* |
|  | BRIN 181 | 49 Y | Male | Urine | *E. coli* | *1* |
|  | BRIN 183 | 65 Y | Male | Urine | *E. coli* | *1* |
|  | BRIN 189 | 50 Y | Male | Urine | *E. coli* | *1* |
|  | BRIN 194 | 53 Y | Female | Urine | *E. coli* | *1* |
|  | BRIN 197 | 37 Y | Female | Urine | *E. coli* | *1* |
|  | BRIN 207 | 75 Y | Male | Urine | *E. coli* | *1* |
|  | BRIN 211 | 48 Y | Female | Urine | *E. coli* | *1* |
|  | BRIN 241 | 60 Y | Male | Urine | *E. coli* | *1* |
|  | BRIN 243 | 56 Y | Male | Urine | *E. coli* | *1* |
|  | BRIN 246 | 38 Y | Male | Urine | *E. coli* | *1* |
|  | BRIN 249 | 60 Y | Male | Urine | *E. coli* | *1* |
|  | BRIN 274 | 61 Y | Female | Urine | *E. coli* | *1* |
|  | BRIN 284 | 40 Y | Female | Urine | *Klebsiella pneumoniae* | *0* |
|  | BRIN 294 | 53 Y | Female | Urine | *E. coli* | *1* |
|  | BRIN 297 | 37 Y | Female | Urine | *E. coli* | *1* |

**^BRIN: Burning infection.^**

**Supplementary Table S6:** **Isolated Microorganisms from Different Samples from Surgery & Dermatology Unit.**

| **S.NO** | **Code** | **Age** | **Gender** | **Sample type** | **MOs** | ***E. coli*** |
| --- | --- | --- | --- | --- | --- | --- |
|  | WDIN 12 | 64 Y | Male | Pus | *Klebsiella pneumoniae* | *0* |
|  | SKIN 25 | 62 Y | Male | Pus | No growth | *0* |
|  | WDIN 33 | 64 Y | Male | Pus | No growth | *0* |
|  | SKIN 38 | 35 Y | Male | Pus | No growth | *0* |
|  | WDIN 60 | 57 Y | Male | Pus | *E. coli* | *1* |
|  | WDIN 79 | 55 Y | Female | Pus | No growth | *0* |
|  | WDIN 102 | 64 Y | Male | Pus | *Klebsiella pneumoniae* | *0* |
|  | SKIN 112 | 65 Y | Male | Pus | No growth | *0* |
|  | WDIN 116 | 58 Y | Male | Pus | *Klebsiella pneumoniae* | *0* |
|  | SKIN 142 | 65 Y | Male | Pus | No growth | *0* |
|  | WDIN 146 | 62 Y | Male | Pus | *Klebsiella pneumoniae* | *0* |
|  | SKIN 152 | 65 Y | Male | Pus | No growth | *0* |
|  | WDIN 162 | 72 Y | Male | Pus | No growth | *0* |
|  | WDIN 180 | 63 Y | Male | Pus | No growth | *0* |
|  | WDIN 209 | 82 Y | Female | Pus | *Klebsiella pneumoniae* | *0* |
|  | WDIN 212 | 64 Y | Female | Pus | *Klebsiella pneumoniae* | *0* |
|  | WDIN 221 | 68 Y | Male | Pus | *Klebsiella pneumoniae* | *0* |
|  | WDIN 226 | 54 Y | Male | Pus | *Candida albicans* | *0* |
|  | WDIN 232 | 64 Y | Male | Pus | *Klebsiella pneumoniae* | *0* |
|  | WDIN 236 | 64 Y | Female | Pus | *Klebsiella pneumoniae* | *0* |
|  | WDIN 252 | 64 Y | Male | Pus | *Klebsiella pneumoniae* | *0* |
|  | WDIN 260 | 70 Y | Male | Pus | *Klebsiella pneumoniae* | *0* |
|  | WDIN 262 | 64 Y | Male | Pus | *Klebsiella pneumoniae* | *0* |
|  | SKIN 272 | 55 Y | Male | Pus | No growth | *0* |

**^WDIN: Wound Infection, SKIN: Skin Infection.^**

**Supplementary Table S7:** **Isolated Microorganisms from Different Samples from ENT & Ophthalmic Units.**

| **S.NO** | **Code** | **Age** | **Gender** | **Sample type** | **MOs** | ***E. coli*** |
| --- | --- | --- | --- | --- | --- | --- |
|  | OPT 2 | 55 Y | Female | Sputum | *Pseudomonas aeruginosa* | *0* |
|  | ENT 13 | 47 Y | Female | Urine | *E. coli* | *1* |
|  | ENT 14 | 66 Y | Male | Sputum | *Candida albicans* | *0* |
|  | ENT 35 | 69 Y | Male | Pus | *Pseudomonas aeruginosa* | *0* |
|  | ENT 54 | 15 D | Male | Pus | *Klebsiella pneumoniae* | *0* |
|  | ENT 103 | 53 Y | Female | Urine | *Candida albicans* | *0* |
|  | ENT 104 | 66 Y | Male | Sputum | *Candida albicans* | *0* |
|  | ENT 118 | 55 Y | Male | Sputum | *Candida albicans* | *0* |
|  | OPT 122 | 45 Y | Male | Sputum | *Pseudomonas aeruginosa* | *0* |
|  | OPT 132 | 55 Y | Female | Sputum | *Pseudomonas aeruginosa* | *0* |
|  | ENT 148 | 48 Y | Female | Sputum | *Candida albicans* | *0* |
|  | ENT 158 | 48 Y | Female | Sputum | *Candida albicans* | *0* |
|  | ENT 164 | 56 Y | Female | Sputum | *Candida albicans* | *0* |
|  | ENT 174 | 22 Y | Male | Pus | *Klebsiella pneumoniae* | *0* |
|  | ENT 204 | 52 Y | Male | Sputum | *Candida albicans* | *0* |
|  | ENT 214 | 66 Y | Male | Sputum | *Candida albicans* | *0* |
|  | ENT 219 | 74 Y | Female | Sputum | *Candida albicans* | *0* |
|  | ENT 224 | 66 Y | Male | Sputum | *Candida albicans* | *0* |
|  | ENT 230 | 66 Y | Male | Sputum | *Candida albicans* | *0* |
|  | ENT 234 | 66 Y | Male | Sputum | *Candida albicans* | *0* |
|  | ENT 254 | 66 Y | Male | Sputum | *Candida albicans* | *0* |
|  | ENT 255 | 62 Y | Female | Sputum | *Candida albicans* | *0* |
|  | ENT 257 | 75 Y | Male | Sputum | *Candida albicans* | *0* |
|  | ENT 263 | 53 Y | Female | Urine | *Candida albicans* | *0* |
|  | ENT 264 | 66 Y | Male | Sputum | *Candida albicans* | *0* |
|  | ENT 282 | 74 Y | Female | Sputum | *Candida albicans* | *0* |
|  | ENT 286 | 74 Y | Female | Sputum | *Candida albicans* | *0* |
|  | ENT 289 | 74 Y | Female | Sputum | *Candida albicans* | *0* |

**^OPT: Ophthalmic, ENT: Ear, Nose & Throat.^**

**Supplementary Table S8:** **Internal Validation Isolates**

| **S.NO** | **Code** | **Department** | **Age** | **Gender** | **Sample type** | **MOs** | ***E. coli*** |
| --- | --- | --- | --- | --- | --- | --- | --- |
|  | UTI 1 | Urology | 60 Y | Female | Urine | *E. coli* | 1 |
|  | UTI 2 | Urology | 18 Y | Female | Urine | *E. coli* | 1 |
|  | BCEM 3 | PICU | 11 Y | Female | Blood | *S. aureus* | 0 |
|  | BCEM 4 | PICU | 9 Y | Female | Blood | No growth | 0 |
|  | UTI 5 | Urology | 50 Y | Male | Urine | *E. coli* | 1 |
|  | HEMU 6 | Urology | 65 Y | Male | Urine Catheter | *C. albicans* | 0 |
|  | UTI 7 | Urology | 40 Y | Female | Urine | *E. coli* | 1 |
|  | BRIN 8 | Burning | 30 Y | Male | Pus | *P. aeruginosa* | 0 |
|  | BRIN 9 | Burning | 15 Y | Male | Pus | *S. aureus* | 0 |
|  | WDIN 10 | Surgical | 17 Y | Male | Pus | *E. coli* | 1 |
|  | BRIN 11 | Burning | 20 Y | Female | Pus | *E. coli* | 1 |
|  | UTI 12 | Urology | 37 Y | Female | Urine | *E. coli* | 1 |
|  | BCEM 13 | ICU | 35 Y | Female | Blood | *S. aureus* | 0 |
|  | BRIN 14 | Burning | 22 Y | Male | Pus | *P. aeruginosa* | 0 |
|  | UTI 15 | Urology | 50 Y | Female | Urine | *E. coli* | 1 |
|  | UTI 16 | Urology | 60 Y | Female | Urine | *E. coli* | 1 |
|  | UTI 17 | Urology | 31 Y | Female | Urine | *E. coli* | 1 |
|  | BRIN 18 | Burning | 20 Y | Female | Pus | *P. aeruginosa* | 0 |
|  | BCEM 19 | ICU | 65 Y | Female | Blood | *E. coli* | 1 |
|  | PNEU 20 | ICU | 35 Y | Female | Blood | No growth | 0 |
|  | PNEU 21 | ICU | 20 Y | Female | Blood | *E. coli* | 1 |
|  | BRIN 22 | Burning | 8 Y | Male | Pus | *P. aeruginosa* | 0 |
|  | BRIN 23 | Burning | 55 Y | Male | Pus | *P. aeruginosa* | 0 |
|  | BCEM 24 | ICU | 20 Y | Female | Blood | *E. coli* | 1 |
|  | WDIN 25 | Surgical | 25 Y | Female | Pus | *S. aureus* | 0 |
|  | WDIN 26 | Surgical | 23 Y | Female | Pus | *E. coli* | 1 |
|  | UTI 27 | Urology | 27 Y | Female | Urine | *E. coli* | 1 |

**Supplementary Table S8 (Continue): Internal Validation Isolates**

| **S.NO** | **Code** | **Department** | **Age** | **Gender** | **Sample type** | **MOs** | ***E. coli*** |
| --- | --- | --- | --- | --- | --- | --- | --- |
| **28** | BCEM 28 | Urology | 18 Y | Female | Blood | *S. aureus* | 0 |
| **29** | PNEU 29 | ICU | 41 Y | Female | Sputum | *K. pneumonia* | 0 |
| **30** | BCEM 30 | ICU | 21 Y | Female | Blood | *E. coli* | 1 |
| **31** | PNEU 31 | ICU | 18 Y | Female | Sputum | *K. pneumonia* | 0 |
| **32** | BCEM 32 | ICU | 56 Y | Female | Blood | *S. aureus* | 0 |
| **33** | BCEM 33 | ICU | 70 Y | Female | Blood | *E. coli* | 1 |
| **34** | UTI 34 | ICU | 65 Y | Male | Urine | No growth | 0 |
| **35** | WDIN 35 | Urology | 72 Y | Male | Pus | *E. coli* | 1 |
| **36** | PNEU 36 | Surgical | 48 Y | Female | Blood | *E. coli* | 1 |
| **37** | UTI 37 | ICU | 35 Y | Female | Urine | *E. coli* | 1 |
| **38** | BRIN 38 | Urology | 32 Y | Female | Blood | *E. coli* | 1 |
| **39** | BRIN 39 | Burning | 38 Y | Female | Blood | No growth | 0 |
| **40** | UTI 40 | Burning | 23 Y | Female | Urine | *E. coli* | 1 |
| **41** | UIRR 41 | Urology | 29 Y | Female | Urine | *E. coli* | 1 |
| **42** | PNEU 42 | Urology | 70 Y | Male | Sputum | *K. pneumonia* | 0 |
| **43** | BCEM 43 | ICU | 62 Y | Male | Body fluid | *S. pneumoniae* | 0 |
| **44** | HEMU 44 | ICU | 45 Y | Female | Urine | *E. coli* | 1 |
| **45** | WDIN 45 | Urology | 22 Y | Female | Blood | *E. coli* | 1 |
| **46** | UIRR 46 | Surgical | 17 Y | Female | Urine | No growth | 0 |
| **47** | WDIN 47 | Urology | 30 Y | Female | Pus | *P. aeruginosa* | 0 |
| **48** | UTI 48 | Surgical | 45 Y | Male | Urine | *E. coli* | 1 |
| **49** | PNEU 49 | Urology | 37 Y | Male | Sputum | *K. pneumonia* | 0 |
| **50** | WDIN 50 | ICU | 22 Y | Female | Pus | *S. aureus* | 0 |
| **51** | BCEM 51 | Surgical | 19 Y | Male | Blood | *E. coli* | 1 |
| **52** | UTI 52 | ICU | 25 Y | Female | Urine | No growth | 0 |
| **53** | UTI 53 | Urology | 27 Y | Female | Urine | *E. coli* | 1 |
| **54** | UTI 54 | Urology | 30 Y | Female | Urine Catheter | *C. albicans* | 0 |

**Supplementary Table S8 (Continue): Internal Validation Isolates**

| **S.NO** | **Code** | **Department** | **Age** | **Gender** | **Sample type** | **MOs** | ***E. coli*** |
| --- | --- | --- | --- | --- | --- | --- | --- |
| **55** | BRIN 55 | Burning | 35 Y | Male | Pus | *P. aeruginosa* | 0 |
| **56** | BRIN 56 | Burning | 60 Y | Male | Blood | *E. coli* | 1 |
| **57** | UTI 57 | Urology | 30 Y | Female | Blood | *E. coli* | 1 |
| **58** | UTI 58 | Urology | 23 Y | Female | Urine | *E. coli* | 1 |
| **59** | BRIN 59 | Burning | 9 Y | Female | Pus | *P. aeruginosa* | 0 |
| **60** | BRIN 60 | Burning | 20 Y | Female | Blood | *E. coli* | 1 |
| **61** | BRIN 61 | Burning | 34 Y | Female | Pus | *P. aeruginosa* | 0 |
| **62** | UTI 62 | Urology | 29 Y | Female | Urine | *E. coli* | 1 |
| **63** | WDIN 63 | Surgical | 47 Y | Male | Pus | *S. aureus* | 0 |
| **64** | WDIN 64 | Surgical | 67 Y | Male | Pus | *S. aureus* | 0 |
| **65** | BRIN 65 | Burning | 10 Y | Female | Pus | *E. coli* | 1 |
| **66** | BRIN 66 | Burning | 22 Y | Female | Pus | *E. coli* | 1 |
| **67** | UTI 67 | PICU | 8 Y | Female | Urine | *E. coli* | 1 |
| **68** | BCEM 68 | ICU | 31 Y | Female | Blood | *E. coli* | 1 |
| **69** | BRIN 69 | Burning | 70 Y | Male | Pus | *S. aureus* | 0 |
| **70** | UTI 70 | Urology | 31 Y | Female | Urine | *E. coli* | 1 |
| **71** | UTI 71 | Urology | 30 Y | Female | Urine | *E. coli* | 1 |
| **72** | UTI 72 | Urology | 65 Y | Female | Urine | *E. coli* | 1 |
| **73** | BRIN 73 | Burning | 50 Y | Male | Pus | *P. aeruginosa* | 0 |
| **74** | UTI 74 | Urology | 70 Y | Male | Urine | *E. coli* | 1 |
| **75** | UTI 75 | Urology | 63 Y | Male | Urine | *E. coli* | 1 |
| **76** | WDIN 76 | Surgical | 15 Y | Female | Blood | *E. coli* | 1 |
| **77** | BRIN 77 | Burning | 22 Y | Female | Pus | *P. aeruginosa* | 0 |
| **78** | BRIN 78 | Burning | 30 Y | Female | Pus | *P. aeruginosa* | 0 |
| **79** | BCEM 79 | ICU | 46 Y | Male | Blood | *E. coli* | 1 |
| **80** | WDIN 80 | Surgical | 52 Y | Male | Pus | *P. aeruginosa* | 0 |
| **81** | WDIN 81 | Surgical | 22 Y | Female | Pus | *P. aeruginosa* | 0 |

**Supplementary Table S8 (Continue): Internal Validation Isolates**

| **S.NO** | **Code** | **Department** | **Age** | **Gender** | **Sample type** | **MOs** | ***E. coli*** |
| --- | --- | --- | --- | --- | --- | --- | --- |
| **82** | BRIN 82 | Burning | 33 Y | Female | Pus | *E. coli* | 1 |
| **83** | UTI 83 | Urology | 29 Y | Female | Urine | *E. coli* | 1 |
| **84** | BRIN 84 | Burning | 27 Y | Female | Pus | *P. aeruginosa* | 0 |
| **85** | BCEM 85 | ICU | 40 Y | Female | Blood | *E. coli* | 1 |
| **86** | WDIN 86 | Surgical | 47 Y | Female | Pus | *E. coli* | 1 |
| **87** | ENT 87 | ENT | 50 Y | Female | Sputum | *S. pyogenes* | 0 |
| **88** | ENT 88 | ENT | 20 Y | Female | Sputum | *S. pyogenes* | 0 |
| **89** | ENT 89 | ENT | 35 Y | Male | Sputum | *Candida albicans* | 0 |
| **90** | ENT 90 | ENT | 23 Y | Female | Sputum | *Candida albicans* | 0 |
| **91** | ENT 91 | ENT | 42 Y | Male | Sputum | *S. pyogenes* | 0 |
| **92** | ENT 92 | ENT | 15 Y | Male | Sputum | *S. pyogenes* | 0 |
| **93** | ENT 93 | ENT | 18 Y | Female | Sputum | *S. pyogenes* | 0 |
| **94** | ENT 94 | ENT | 29 Y | Female | Sputum | *S. pyogenes* | 0 |
| **95** | ENT 95 | ENT | 50 Y | Male | Sputum | *Candida albicans* | 0 |
| **96** | ENT 96 | ENT | 27 Y | Male | Sputum | *Candida albicans* | 0 |
| **97** | ENT 97 | ENT | 15 Y | Female | Sputum | *Candida albicans* | 0 |
| **98** | ENT 98 | ENT | 10 Y | Female | Sputum | *Candida albicans* | 0 |
| **99** | ENT 99 | ENT | 14 Y | Male | Sputum | *S. pyogenes* | 0 |
| **100** | ENT 100 | ENT | 13 Y | Female | Sputum | *S. pyogenes* | 0 |

**^PNEU: Pneumonia, BCEM: Bacteremia, UTI: Urinary Tract Infection, HEMU: Hematuria, PNEU: Pneumonia, UIRR: Urination Irregular, ENT: Ear, Nose & Throat, WDIN: Wound Infection, BRIN: Burning Infection.^**
